# Supplementary material for: Quantitative evaluation of protective antibody response induced by hepatitis E vaccine in humans
Source: Nat Commun. 2020 Aug 7;11:3971. doi: 10.1038/s41467-020-17737-w (PMC7414844; doi:10.1038/s41467-020-17737-w)
Supplement: Supplementary file 1 — Supplementary Information [file 41467_2020_17737_MOESM1_ESM.pdf]

Supplementary Information

Quantitative evaluation of protective antibody response induced by hepatitis E vaccine in humans

Wen et al

This PDF file includes:

Supplementary Tables 1 to 9

Supplementary Figures. 1 to 18

Supplementary Table 1. Donor information and isolation of HEV p239(1)-specific monoclonal antibodies (mAbs) from four donors.

	Donor 1	Donor 2	Donor 3	Donor 4
Sex	Female	Male	Male	Female
Age	24	19	24	25
Starting PBMCs	~5.0×10^6	~5.0×10^6	~5.0×10^6	~5.0×10^6
HEV p239(1)-sorted B cells	67	55	64	56
Cloned antibodies	54	40	32	37
IgG-expressing clones	45	32	30	30
HEV p239(1)-specific mAbs	32	23	28	21

Fifty-five to 67 HEV p239(1)-specific B cells from each donor were sorted into individual wells, with their Ig gene transcripts recovered by single-cell polymerase chain reaction (PCR). Subsequently, 32 to 54 cognate heavy and light chain pairs from each donor were cloned into the IgG1 vector and expressed as mAbs in ExpiCHO cells. A total of 104 HEV p239(1)-specific mAbs, 21 to 32 from each donor, were obtained.

Supplementary Table 2. Sequence information of HEV p239(1)-specific mAb heavy chains.

ID	Donor	VH germline gene and allele	CDRH3 Sequence	CDRH3 length (aa)	Amino acid sequence of variable domain
A103	Donor 1	IGHV7-4-1*02	AREELGDYHYYMDV	14	QVQLVQSGSELKKPGASVKVSCKASGYTFTDYALNWVRQAPGQGPEWMGWINTNTGNPKYAQGFTGRFVFSLDTSVSTAYLQISSLKAEDTAVYYCAREELGDYHYYMDVWGKGTTVTVSS
A108	Donor 1	IGHV4-39*07	ARDVVDVVATGGGNYYSSYGTDA	23	QVQLQESGPGLVKPSETLSLTCTVSGGSIHSSSYYWGWIRQPPGKRLEWIGNIYYIGSTHYNPSLKSRVTISLDTSRNQFSLKLSSVTAADTAVYYCARDVVDVVATGGGNYYSSYGTDAWGQGTTVTVSS
A109	Donor 1	IGHV3-48*04	ARTRGSLVPGLTYYMDV	17	EVQLVESGGGLVQPGGSLRLSCAVSGFTFSSYSMNWVRQAPGKGLEWVSYIGSSSSTIFYADSVKGRFTISRDNAKNSLYLQMNSLRAEDTAMYYCARTRGSLVPGLTYYMDVWGKGTTVTVSS
A113	Donor 1	IGHV1-8*02	ARVPTDDSSGYWMYLDL	17	QVQLVQSGAEVKKPGASVKVSCKASGYTFTSYDINWVRQATGQGLEWMGWMNPNSGNAGYAQKFQGRVTMTRNISISTAYMELSSLRSEDTAVYYCARVPTDDSSGYWMYLDLWGRGTLVTVSS
A115	Donor 1	IGHV1-8*02	ARVPTDDSSGYWMYFDL	17	QVQLVQSGAEVKKPGASVKVSCKTSGYTFTSYDINWVRQATGQGLEWMGWMNPNSGNADYAQRFQGRVTMTRNTSISTAYMELSSLRSEDTAVYYCARVPTDDSSGYWMYFDLWGRGTLVTVSS
A130	Donor 1	IGHV3-30*04	ARDGGSRSDPIDH	13	EVQLVESGGGVVQPGRSLRLSCAASGFTFSTYAMHWVRQAPGKGLKWVAVISYDGSNKYYADSVKGRSTISRDNSKNTLYLQMNSLRAEDTAVYYCARDGGSRSDPIDHWGQGTLVTVSS
A219	Donor 1	IGHV1-69*01	ARYSTPDRGRFYYYHGLDV	19	QVQLVQSGAEVKKPGSSMKVSCQASGGTFSDFAINWVRQAPGQGLEWMGGIIPLFGTLNYAQKFQGRVTITADESTNTVYMQLSSLRSEDTAVYYCARYSTPDRGRFYYYHGLDVWGQGTTVTVSS
A221	Donor 1	IGHV4-38-2*02	ARDCRVVPARPYYYSYMDV	19	QVQLQESGPVLVKSSETLSLTCTVSGSSISSGYYWGWIRQPPGKGLEWIGAIYNTESTYYNPSLKSRVTISGDSSKNQFSLKLRSVTAADTAVYYCARDCRVVPARPYYYSYMDVWGKGTTVTVSS
A223	Donor 1	IGHV4-59*01	ARETLSTYGYGLYFDS	16	QVQLQESGPGLVKPSETLSLTCTVSGGSINSYYWSWIRQPPGKGLEWIGYIFYSGITNYNVSLKSRVTMSVDTSKNQFSLRLRSVTAADTAVYYCARETLSTYGYGLYFDSWGQGTLVTVSS
A224	Donor 1	IGHV3-43D*01	AKGRDGDHVGFYNYGMDA	18	EVQLVESGGVVVQPGGSLRLSCAASGFTFDDYAMQWVRQPPGKGLEWVSLISWDGHYTFYADSVKGRFTISRDNRKKSLYLQINSLRPEDTALYYCAKGRDGDHVGFYNYGMDAWGQGTTVTVSS
A225	Donor 1	IGHV1-69*01	ATRRDTSMVNYYYYGMDV	18	QVQLVQSGAEVKKPGSSVKVSCKASGGTFNSYVFSWVRQAPGQGLEWMGGIIAVFGTVDYAQKFQGRVTITADDSTSTAYMELSSLRSEDTAVYYCATRRDTSMVNYYYYGMDVWGQGTTVTVSS
A231	Donor 1	IGHV3-21*01	ARAGSSYHYYYMDV	15	EVQLAESGGGLVKPGGSLRLSCAASGFTFSNYSINWVRQAPGKGLEWVSCITDSSRYIFYADSVKGRFTISRDNAKNSLYLQMNSLRAEDTAIYYCARAGSSYHYYYYMDVWGKGTTVTVSS
A236	Donor 1	IGHV1-69*01	ARDRDSYYAEGSSGLDV	17	QVQLVQSGAEVKKPGSSVKVSCKASGGTFASYGISWVRQAPGQGLEWMGGIIPLFGNPNYAQKFQGRVTITADESTSTAYMELSSLRSEDTAVYYCARDRDSYYAEGSSGLDVWGQGTTVSVSS
A239	Donor 1	IGHV1-18*01	ARGDFYFYGMDV	12	QVQLVQSGAEVKKPGASVKVSCKASGYSFTSYYINWVRQAPGQGLEWVGWISSYNGNTNYAEKLQGRVTMTTDTSTSTAYMELRSLRSDDTAVYYCARGDFYFYGMDVWGQGTTVTVSS
A253	Donor 1	IGHV3-11*05	ANWRSEVPAVLEKDAFDI	18	QVQLVESGGGLVKPGGSLRLSCAASGFTFSDYYMSWIRQAPEKGLEWVSYISSSSSYTNYADSVKGRFTISRDNTKNSLYLQMNSLRAEDTAVYYCANWRSEVPAVLEKDAFDIWGQGTMVTVSS
A254	Donor 1	IGHV4-31*03	ARERGISHTTTSRADYFDY	19	QVQLQESGPGLVKPSQTLSLTCTVSGGSISSGGYYWNWIRQHPGKGLEWIGYISYSGSTYYNPSLRSRLSISVDTSKSHFSLKLRSVTAADTAVYYCARERGISHTTTSRADYFDYWGQGTLVTVSS
A255	Donor 1	IGHV1-69*06	AKEPLTDFWSGYWAY	15	QVQLVQSGAEVKKPGSSVKVSCKASGGTSSTYGINWVRQAPGQGLEWMGGLIPILGTANFAQKFQDRVTITADTSTTTVYMELSSLRYEDTAVYYCAKEPLTDFWSGYWAYWGQGSLVTVSS
A257	Donor 1	IGHV7-4-1*02	ARDGISNPYRQNPVAGMDWELLWDY	25	QVQLVQSGSELKKPGASVKVSCEASGYTFTSFAMNWVRQAPGQGLEWVGWINTHTGNPTYAQGFTGRFVFSLDTSVSTAYLQISSLKAEDTAVYYCARDGISNPYRQNPVAGMDWELLWDYWGQGTLVTVSS
A258	Donor 1	IGHV1-18*01	ARAHLVVAGRHHFHGMDV	18	QVKLVQSGGEVKKPGASVKVSCKASGYTFSTYGISWVRQAPGQGPEWMGWISAYNGDTTYAQKFQGRVTMTTDTSTMTAFMDLGSLTSDDTAVYYCARAHLVVAGRHHFHGMDVWGQGTTVTVSS
A260	Donor 1	IGHV1-69*01	ARGGDYGGNPYYFDD	15	QVQLVQSGAEVKKPRSSVKVSCKASGGTFSSSAFTWVRQAPGQGLEWMGGIFTLFQTVNSAQKFQGRITITADESTNTAYMELSNLKSEDTAVYYCARGGDYGGNPYYFDDWGQGTLVTVSS
A266	Donor 1	IGHV4-59*08	ARFRNSRWYEAGFDI	15	QVQLQESGPGLVKPSETLSLICSVSGDSISSYYWSWIRQPPGKGLEWIGYIYYSGISNYNPSLKSRVTMSVETSKNQVSLRLYSVTGADTAVYYCARFRNSRWYEAGFDIWGQGTMVTVSS
A269	Donor 1	IGHV4-39*07	ARDVVDVVATGGGNYYFFYGTDV	23	QVQLQESGPGLMRPSETLSLTCTVSGGSVSSSTHYWAWIRQPPGKGLEWIANIYYSGSANYNPSLRSRVAISLDTSKNQFSLQLTSVTAADTAVYYCARDVVDVVATGGGNYYFFYGTDVWGHGTTVTVSS
A271	Donor 1	IGHV4-61*08	ARDYNAMDV	9	QVQLQESGPGLVKPSETLSLTCTVSGGSLSSRGYYWSWIRQPPEKGLEWIGYIHYSGSTNYNPSLKSRVTISIDTSKNQFSLRLRSVTAADTALYYCARDYNAMDVWGQGTTVTVSS
A273	Donor 1	IGHV1-69*03	CARHQGHGQRSSWWDYYYYDMDV	23	QVQLVQSGAEVKKPGSSVKVSCRASGGTFSSYAISWVRQAPGQGLEWMGGIIPIFVTSNHYAQKFQGRVTMTADESTSTAYMELSSLRSEDTAVYYCARHQGHGQRSSWWDYYYYDMDVWGQGTTVTVSS
A275	Donor 1	IGHV4-59*01	ARVSGCGGYCYLGAAIDY	18	QVQLQESGPGLVKPSETLSLTCTVSRDSISTYYWTWIRQPPGKGLEWIGFIYHTGSTNYNPSLKSRVTMSIDTSRDQFSLRLTSVTSADTAVYYCARVSGCGGYCYLGAAIDYWGQGTLVTVSS

A278	Donor 1	IGHV1-69*01	ATEGGSAWNDHGFEI	15	QVQLVQSGAEVKKPGSSVKVSCKASGGTFNGYSVNWVRQAPGQGLEWMGGIIPIFGTANYAQKFQDRVTITADESTSTAYMELRSMRSEDTAVYYCATEGGSAWNDHGFEIWGQGTMVTVSS
A280	Donor 1	IGHV3-11*05	ATWRSEGPAALEKDAFDI	18	EVQLVESGGGVVKPGGSLRLSCAASGFPFSDYYMSWIRQAPGKGLEWVSYISSISVYTNYADSVKGRFTISRDNAKNSLYLQMNNLRAEDTAVYYCATWRSEGPAALEKDAFDIWGQGTMVTVSS
A283	Donor 1	IGHV4-39*06	ARDVVDIVATGGGNHYFYYGTDV	23	SRTPQESGPGLVKPSETLSLTCTVSGGSISTSTYYWGWIRQPPGKGLEWIGRIHYSGSTYYNPSLRSRVTISADTSKNHFSLKLSSVTAADTAVYYCARDVVDIVATGGGNHYFYYGTDVWGQGTTVTVSS
A284	Donor 1	IGHV4-4*07	ARDVEYFDY	9	QVQLQESGPGLVKPSETLSLTCTVSGGSISSHYWSWIRQPAGKGLEWIGRVYPGDITNYNPSLKSRVTMSVDTSKNQFSLKLNSVTAADTAVYYCARDVEYFDYWGQGILVTVSS
A285	Donor 1	IGHV4-4*07	ARDVEFFDS	9	QVQLQESGPGLVKPSETLSLTCTVSGGSISTYYWSWIRQPAGKGLEWIGRIYTNDITNYNPSLKSRATMSVDTSKNQFSLRLNFVTAADTAVYYCARDVEFFDSWGQGTLVTVSS
A286	Donor 1	IGHV3-30*03	ARRRGHCSQTSCYMDP	16	EVQLVESGGGVVQPGRSLRLSCVVSGLSVISSGIYWVRQCPGKGLEWVTLLSDDERNKYYADSVKGRFTISRDNSKNTVYLQMNSLRAEDTGVYYCARRRGHCSQTSCYMDPWGQGTLVTVSA
A287	Donor 1	IGHV1-69*01	ARGEGHNWNVQYYFHH	16	QVQLVQSGAEVKKPGSSVKVSCKASGGTFINYAFNWVRQAPGQGLEWMGGIIPMFATANYAQQFQGRVTITADESSSTAYMELSSLRSEDTAVYYCARGEGHNWNVQYYFHHWGQGTLVTVSS
B103	Donor 2	IGHV5-51*03	ASIAIYYFDSSGSYHRPDAFDI	22	EVQLVQSGAEVKKPGESLKISCKGSGYSFTSYWIGWARQMPGKGLEWMGIIYPGDSDTRYSPSFQGQVSISADKSINTAYLQWSSLKASDTAMYYCASIAIYYFDSSGSYHRPDAFDIWGQGTMVTVSS
B107	Donor 2	IGHV5-51*01	AVTRESIADRTDAFDI	16	EVQLVQSGAEVKEPGESLKMSCKASGYSFPSFWIGWVRQMPGKGLEWMGIIYPGDSDTRYSPSFQGRVTLSVDKSISTAYLQWSSLKASDTATYYCAVTRESIADRTDAFDIWGQGTVVTVSS
B112	Donor 2	IGHV1-69*01	ARDGAYSSGNPWLMAYYFDY	20	QVQLVQSGAEVKKPGSSVKVSCKASGGTFSTSAINWVRQAPGQGLEWMGGIIPIFVTANYAQQFQGRVTITADESTSTAYMELSSLRSEDTAVYYCARDGAYSSGNPWLMAYYFDYWGQGTLVTVSS
B113	Donor 2	IGHV1-69*01	TRDAGGFDP	9	QVQLVQSGAEVKKPGSSVKVSCKASGGSFNTYSINWVRQAPGQGLEWVGGMIPIFGTAKYAQNLQGRVTITADESTSTVFLELSSLRSEDTAVYYCTRDAGGFDPWGQGTLVTVSS
B117	Donor 2	IGHV1-69*01	ARADTPMAHYYYAMDV	16	QVQLVQSGAEVKRPGSSVKVSCKASGGTFSSHAITWVRQAPGQGLEYMGRIIPIFGTTNYAQKFQGRATITADESTNTVYMELSSLRSEDTAVYYCARADTPMAHYYYAMDVWGQGTTV
B121	Donor 2	IGHV1-69*01	ARAGGNTNGNPWHVAYYFEY	20	QVQLVQSGTEVKKPGSSVKVSCEASGGTFNNYAINWVRQAPGEGLEWMGGIIPLFGSASYAQNFQDRVTITADESTGTAYMELTSLKSEDTAIYYCARAGGNTNGNPWHVAYYFEYWGQGTLVTVSS
B125	Donor 2	IGHV1-69*01	AREICNRTSCHWSPELRHMDV	21	QVQLVQSGAEVKKPGSSVKLSCKASGGTFTFYAISWVRQAPGQGLEWMGGFIPLFHTGNYAQKFQGRVTITADESTSTAFMELSSLRSEDTAVYYCAREICNRTSCHWSPELRHMDVWGQGTTVTVSS
B128	Donor 2	IGHV1-69*01	ANHDSDYYYYSMDV	14	QVQLVQSGAEVKKPGSSVKVSCKASGGTFSNYAISWVRQAPGHGLEWMGGIIPIFHIPNYAQKFQGRVTITADESTSTAYMELSGLRSDDTAVYYCANHDSDYYYYSMDVWGQGTAVIVSS
B131	Donor 2	IGHV4-4*07	ARDLGPRWLGELSDPLNWFDP	21	QVQLQESGPGLVKPSETLSLTCTVSGDSISNYYWSWIRQPAGKGLEWIGRVYSSGSTNYNPSLKSRVTMSEDTSKNQFSLKLSSVTAADTAVYYCARDLGPRWLGELSDPLNWFDPWGQGILVTVSS
B137	Donor 2	IGHV3-30*04	ARDVPWRSSSHITNYFDN	18	EVQLVESGGGVVQPGRSLRLSCAASGFTFSYYNMSWVRQAPGKGLEWVALISHDGSHEYYLDSVKARFTISRDNSNNTLCLQMNSLRGEDTAVYYCARDVPWRSSSHITNYFDNWGLGTLVTVAS
B142	Donor 2	IGHV1-69*02	AGDKPSIGGDVHRSLWFGEFFELES	25	QVQLVQSGAEVKKPGSSVKVSCKASGGSFSSYTISWVRQAPGHGLEWMGKIIPIVDIANYAQKFQGRVTITADKSTSTAYMELNSLRSEDTAVYYCAGDKPSIGGDVHRSLWFGEFFELESWGQGTLVTVSS
B143	Donor 2	IGHV4-61*01	AREVVDIVATGQNDYYYYYGMDV	23	QVQLQESGPGLVKPSETLSLTCTVSGGSVSSGSSHWSWIRQSPEKGLEWIGYIHYTGSTKYNPSLKSRVTIFVDTSKNQFSLRLSSVTAADTAVYYCAREVVDIVATGQNDYYYYYGMDVWGQGTTVTVSS
B146	Donor 2	IGHV3-15*01	TIEHAEMAPGGFYYYGMDV	19	EVQLVESGGGLVKPGGSLRLSCAASGFSFSHAWMTWVRQAPGKGLEWVGRIKSKIDGGTTDYAAPVKGRFIISRDDSKNTLYLHMNSLKTEDTAMYYCTIEHAEMAPGGFYYYGMDVWGQGTTVTVSS
B148	Donor 2	IGHV5-51*03	ARLGEGGECPPCGYSYGLNV	20	EVQLVQSGTEVKKPGESLKISCKGSGYSFISYWIGWVRQMPGKGLEWMAIIYPGDSDTRYSPSFQGQVTISADKSISTAYLQWSSLKASDTAMYYCARLGEGGECPPCGYSYGLNVWGQGTTVTVSS
B150	Donor 2	IGHV5-10-1*03	ARHGIHSDLWSGYYDGGFDP	20	EVQLVQSGAEVKKPGESLRISCKGSGYSFTSYWISWVRQMPGKGLEWMGRIDPSDSYTNYSPSFQGHVTISADKSINTAYLQWSSLKASDSAMFYCARHGIHSDLWSGYYDGGFDPWGQGTLVTVSS
B155	Donor 2	IGHV3-23*01	ARDGIVWGNLEWFPCYFDC	19	EVQLLESGGGLVQPGGSLRLSCATSGFTFSSYAMSWVRQAPGKGLEWVSKISGSSGSTDYADSVKGRFTISRDNSKNTLYLQMNSLRADDTAVYYCARDGIVWGNLEWFPCYFDCWGQGTLVTVSS
B157	Donor 2	IGHV1-69*01	ARDRERHSWADHHYGMDV	18	QVQLVQSGAEVKKLGSSVKVSCKASGGTFSTYSINWVRQAPGQGLEWMGGIIPIFGTPNYARKFQGRVTITADESTSTAYMELSSLISEDTAIYYCARDRERHSWADHHYGMDVWGQGTTVTVSS
B164	Donor 2	IGHV5-51*01	ARRVDFPYCGGDCPNWFDP	19	EVQLVQSGAEVKKPGESLKISCKGSGYSFTSYWIGWVRQMPGKGLEWMGIIYPGDSDTRYSPSFQGQVTISADKSISTAYLQWSSLKASDTAMYYCARRVDFPYCGGDCPNWFDPWGQGTLVTVSS
B167	Donor 2	IGHV1-18*01	ARYNVDTVATGGGDYYYYYGMDV	23	QVQLVQSGAEVKKPGASVKVSCKASGFTFSSYGISWVRQAPGQGLEWMGWIGAYNGNTNYAQKLQGRITMTTDTSTNTAYMELRSLRSDDTAVYYCARYNVDTVATGGGDYYYYYGMDVWGQGTTVTVSS
B170	Donor 2	IGHV1-69*01	ARGGTGNPWRDAYYYGMDV	19	QVQLVQSGAEVKKPGSSVKVSCQASGGTFTTYAISWVRQAPGQGLEWMGGIIPIFVTANYAQKFQGRVTITADESTNTAYMELSSLRSEDTAVYYCARGGTGNPWRDAYYYGMDVWGQGTTVTVSS

B173	Donor 2	IGHV1-18*04	ARHLMSVGEWEVFLGFDY	18	QVQLVQSGAEVKKPGASVKVSCKASGYTFSTYGISWVRQAPGQGLEWLGWVSAYNGNTNYAQNLQGRVTMTTETSTSTAYMELRSLRSDDTAVYYCARHLMSVGEWEVFLGFDYWGQGTLVTVSS
B178	Donor 2	IGHV5-51*01	AKHEAVAVAGTDYYYFGMDV	20	EVQLVQSGAEVKKPGESLKISCKGSGYSFTSYWIGWVRQMPGKGLEWMGIIYPGDSETRYSPSFQGQVTISADKSISTAYLQWSSLKASDTAMYYCAKHEAVAVAGTDYYYFGMDVWGQGTTVTVSS
B193	Donor 2	IGHV1-69*01	ARENAPYTYGYYNWFDP	17	QVQLVQSGAEVKKPGSSVKVSCKASGGTFSSHAINWVRQAPGQGLEWMGGIIPIFGTVNYAQKFQGRVTITADESTNTAYMEVNSLRSEDTAVYYCARENAPYTYGYYNWFDPWGQGTLVTVSS
C6	Donor 3	IGHV4-31*03	ARDRGSIVEPAALYIDY	17	QVQLVESGGGVVQPGRSLRLSCAASGFTFRSYAIHWVRQAPGKGLEWVALISYDGSNGYYADSVKGRFTISRDNSKNTVYLQVNTLRAEDTALYYCARDRGSIVEPAALYIDYWGQGTLVTVSS
C11	Donor 3	IGHV1-18*01	ARSSHYWVPNWFDP	14	QVQLQESGPGLVKPSQTLSLTCTVSGDSINSGGYYWSWIRQHPGKGLEWIGYISYSGSTSYNPSLESRVTISMGTSESQFSLKLRSVTAADTAVYYCARSSHYWVPNWFDPWGQGSLVIVSS
C2F8	Donor 3	IGHV3-11*04	VRGEYHYGSGPDY	13	QVQLVQSGTEVKKPGASVKVSCRASGYSFTTYGITWVRQAPGQGLEWMGWISTFNGNKNFAQKFQGRFTMTTDTSTSTAYMELRSLRSDDTAVYYCVRGEYHYGSGPDYWGQGTLVTVSS
C3C6	Donor 3	IGHV4-4*02	ARSGRTGDAFDI	12	EVQLLESGGGLVKPGGSLRLSCAASGFSFSDYYMTWIRQAPGKGLEWVSYISSSGYTIYYADSVKGRFTISRDNAKNSLYLQMNSLRAEDTAVYYCARSGRTGDAFDIWGQGTMVTVSS
C4D11	Donor 3	IGHV3-30*04	AREGGDAFDI	10	QVQLQESGPGLVKPSGTLSLTCAVSGGSISNVNWWSWVRQPPGKGLEWIGYISYSGSTNYHPSLKSRVSISMNKSTNQFSLQLSSVTAADTAVYFCAREGGDAFDIWGQGTMITVSP
C101	Donor 3	IGHV1-69*01	ASGSGDYYTKGYYFEY	16	QVQLVQSGAEVKKTGSSVKVSCKASGGTLNNFAFNWVRQAPGQGPEWMGGIIPSVGVPNYTLKLQGRVSITADASTSTAYMELSSLGSEDTAVYYCASGSGDYYTKGYYFEYWGQGTLVTVSS
C104	Donor 3	IGHV1-8*01	ARGRGGVVSPAAIYMDV	17	QVQLVQSGAEVKKPGASVKVSCKASGYTFTGYDIHWVRQATGQGLEWMGWMIPNSGNTGYAQKFQGRVTMTRNTAISTAYMELSSLRSDDTAVYYCARGRGGVVSPAAIYMDVWGKGTTVTVSS
C109	Donor 3	IGHV1-46*01	ARDGGGQQLLKLDY	14	QVQLVQSGAEVKKPGASVMVSCKASGYTFTTYYVHWVRQAPGQGLEWMGTINPSGGSTGYSQKFQGRVTMTSDTSTNTVYVELSGLTSDDTALYYCARDGGGQQLLKLDYWGQGTLVTVSS
C113	Donor 3	IGHV1-69*01	ARNRVKWNDLFCMDV	15	QVHLVQSGAEVKKPGSSVKVSCKASGGTFSTSGISWVRQAPGQGLEWMGAIIPIFTTANYAQNFQGRVTITADESTNTAYMELSSLRSEDTAVYYCARNRVKWNDLFCMDVWGEGTTVTVSS
C122	Donor 3	IGHV7-4-1*02	AREVPGIAYYYYYMDV	16	QVQLVQSGSELKKPGASVKVSCKASGYTFTNYAVNWVRQAPGQGLEWMGWINTNNGNPTYAQGFTGRFVFSLDTSVSTAYLQISSLKAEDTAMYYCAREVPGIAYYYYYMDVWGKGTAVTVSS
C127	Donor 3	IGHV1-18*01	ARYRGSTVVPAAIVFDF	17	QVQLVQSGTELKKPGASVTVSCQASGYTFTRYGVSWMRQAPGQGLEWMGWISVHNGHTTYSQSVQGRVTVTTDTSTNTAYMTLRGLRTDDTAVYYCARYRGSTVVPAAIVFDFWGQGTLVTVSS
C131	Donor 3	IGHV1-69*01	AREAQPNPWFRENNRFDP	18	QVQLVQSGAEVKKPGSSVKVSCKTSGGTFSTYGISWVRQAPGQGLEWLGGIIPIFATPNYAQNFQGRLTITADESTSTAYMELTSLRSDDTAVYYCAREAQPNPWFRENNRFDPWGQGTLVTVSS
C136	Donor 3	IGHV1-8*01	ARGRYYEFSSGPVPDDGFDI	20	QVLLVQSGAEVKKPGASVKVSCRASGYTFTSYDIHWVRQAAGQGLEWMGWMNPNSGDTGSAQKFQGRVTMTRDTSISTAYMELSSLNSEDTAVYYCARGRYYEFSSGPVPDDGFDIWGQGTMVTVSS
C139	Donor 3	IGHV1-18*01	ARDGYNYRDAFDI	13	QVQLVQSGAEVKKPGASVKVSCKASGYTFTTYGISWVRQAPGQGLEWMGWISAYNDDTNYAQKFQGRVTMTTDTSTSTAYMELRSLRSDDTAVYYCARDGYNYRDAFDIWGQGTKVTVSS
C142	Donor 3	IGHV1-58*02	AAQTYYYDGSGYYSVSEFDP	20	QMQLVQSGPEVRKPGTSVKVSCKASGFPFSNSAIQWMRQTRGQRLEWIGWVVVGGGDTNYAQNFQDRVTITTDMSTATAYMELNSLRSEDTAVYYCAAQTYYYDGSGYYSVSEFDPWGQGTLVTVSS
C144	Donor 3	IGHV1-69*01	ARDLRSSSSWHSNYYMDV	18	QVQLVQSGAEVKKPGSSVKVSCKASGGTFSTSAISWVRQAPGQGLEWMGGIIPIFVTPNYAQKFQGRVTITADESTNTAYMELSSLRSEDTAVYYCARDLRSSSSWHSNYYMDVWGKGTTVTVSS
C145	Donor 3	IGHV1-8*01	ARGRGTTRFSLQNFPFDNAYYMDV	24	QVQLVQSGAEVKKPGASVKVSCETSGYTFTSYNINWVRQATGQGLEWMGWMNPTDGNTDYAQKFQGRVSMTRDTSISTAYMELSSLRSEDTAVYYCARGRGTTRFSLQNFPFDNAYYMDVWGKGTTVTVSS
C150	Donor 3	IGHV1-8*01	ARAKSGDYYYHMDI	14	QVQLVQSGAEVKKPGASVKVSCKTSGYTFTNYEIHWVRQATGQGLEWMGWMNPNSGNKGYTQKFQGRVTMTRNTSINIAYMELSSLRSEDTAVYFCARAKSGDYYYHMDIWGKGTTVTVSS
C158	Donor 3	IGHV1-3*01	ARERGGSVVEPAAHYMDV	18	QVQIVQSGAEVKKPGASVKVSCTASGYTFTRYGLVWVRQAPGQGLEWMGSINTGNANTIYSEKFQGRVSITRDTSASTTYMELRSLRYEDTAVYFCARERGGSVVEPAAHYMDVWGNGTTVSVTS
C168	Donor 3	IGHV1-69*01	ARDGSSSPWQTNYYNMDV	18	QVQLVQSGPEVKKPGSSVKVSCKASGGTFSTSAISWVRQAPGQGLEWMGGIIPIFYTPNYAQKFQGRVTISADESTSTAYMELSSLMSEDTAVYYCARDGSSSPWQTNYYNMDVWGDGTTVIVSS
C169	Donor 3	IGHV1-8*01	ARGRGRVGRYLERLLSYYMDV	21	QVQLVQSGAEVKKPGASVKVSCKASEHTFTRYDITWVRQATGQGLEWMGWMNPKSGNTGYAQKFQGRVTMSRNTSITTAYMELSSLTSEDTAVYYCARGRGRVGRYLERLLSYYMDVWGNGTTVTVSS
C172	Donor 3	IGHV1-18*01	ARETTMTADPRYYSYYYMDV	20	QVQLVQSGAEVKKPGASVKVSCRASGYRFTSYGVNWVRQAPGQGLEWMGWISGYNGNKNYAQKFQGRVTLTTDTSTTTAYMELRSLRSDDTAVYYCARETTMTADPRYYSYYYMDVWGTGTTVTVSS
C182	Donor 3	IGHV1-18*01	ARDLLTLGYCSTFSCSTPLDY	21	QVQLVQSGAEVKKPGASVKVSCKASGYTFSSYGISWVRQAPGQGLEWMGWISGYNGNKNYAQKVQGRVTLTTDTSTSTAYMELRSLRSDDTAVYYCARDLLTLGYCSTFSCSTPLDYWGQGTPVTVSS
C183	Donor 3	IGHV1-3*01	ARERGGSVVVPAAHYMDV	18	QVQLVQSGAEVKKPGASVKVSCKASGYTFTRFAMVWVRQAPGQRLEWMGWINAGNGNTKYSQKFQGRVSMTRDTSASTAYMELSSLRSEDSAVYYCARERGGSVVVPAAHYMDVWGKGTTVTVTS

C3E10	Donor 3	IGHV4-61*01	ARNYRRTSISSPNYYFYMDV	20	QVQLQESGPGLVKPSETLSLTCTVSGDSINSGSHYWSWIRQSPGKGLDWIGYIYHSGSTNYNPSLKSRVTISVDTSKNQFSLRLNSVTAADTAVYYCARNYRRTSISSPNYYFYMDVWGRGTTVTVSS
C3G2	Donor 3	IGHV1-69*01	AGEVGAPRIQWFREVGWFDP	20	QVQLVQSGAEVKKPGSSVKVSCKASGGTFSSYAISWVRQAPGQGLEWMGGIIPIFGTANYAQKFQNRVTITADESTSTAYMELSSLRSEDTAVYYCAGEVGAPRIQWFREVGWFDPWGQGTLVTVSS
C4B11	Donor 3	IGHV3-9*01	AKDGSGIPDYYFFYMDV	17	EVQLVESGGGSVQPGRSLRLSCAASGFTFDVHAMHWVRQAPGKGLEWVSGINWDSSRIGYADSVKGRFTISRDNAKNSLYLQMNSLRSEDTALYFCAKDGSGIPDYYFFYMDVWGKGTTVTVSS
C4G10	Donor 3	IGHV1-8*01	AAARRGDYYYYMDV	14	QVQLVQSGAEVEKPGASVKVSCKASGYTFTSYDINWVRQATGQGLEWMGWMNPTSGNRGYAQKYQGRVTMTRNTTISTAYMELSSLRSEDTAVYYCAAARRGDYYYYMDVWGKGTTVTVSS
D115	Donor 4	IGHV3-74*01	ARVGEERYSGYPGSDY	16	EVQLVESGGGLVQPGGSLRLSCAASGFTFSSSWMNWVRQAPGKGLVWVSRINSDGSATIYADSVKGRFTVSRDNAKNTLYLQMNSLRAEDTAVYYCARVGEERYSGYPGSDYWGQGTLVTVSS
D214	Donor 4	IGHV1-69*12	ARNRVKWNDLFCMDV	15	AHSQVQSGAEVKKPGSSVKVSCKASGGTFSTSGISWVRQAPGQGLEWMGAIIPIFTTANYAQNFQGRVTITADESTNTAYMELSSLRSEDTAVYYCARNRVKWNDLFCMDVWGEGTTVTVSS
D242	Donor 4	IGHV1-69*01	AGEEGAPRIQWFREVGWFDP	20	QVQLVQSGAEVKKPGSSVKVSCKASGGTFSSYAISWVRQAPGQGLEWMGGIIPIFGTANYAQKFQNRVTITADESTSTAYMELSSLRSEDTAVYYCAGEEGAPRIQWFREVGWFDPWGQGTLVTVSS
D301	Donor 4	IGHV3-30*03	AKGQIPYDIGHYSMDV	16	EVQLVESGGGVVQPGRSLRLSCAASGFTFTTYGIHWVRQAPGKGLEWVAVISYDGTNKYYADSVKGRFTISRDNSKDTLFLRINSLRVEDTAVYYCAKGQIPYDIGHYSMDVWGQGTTVTVSS
D305	Donor 4	IGHV1-69*01	ARNRVKWNDLFCMDV	15	QVHLVQSGAEVKKPGSSVKVSCKASGGTFSTSGISWVRQAPGQGLEWMGAIIPIFTTANYAQNFQGRVTITADESTNTAYMELSSLRSEDTAVYYCARNRVKWNDLFCMDVWGEGTTVTVSS
D311	Donor 4	IGHV1-69*03	CAREDGSSWDAYYYGMDV	18	QVQLVQSGAEVKKPGSSVRVSCKASGGTFSTSAISWVRQAPGQGLEWMGGIIPIFHTANYAQKFQGRVMIQTADESTSTAYMELSSLRSEDTAVYYCAREDGSSWDAYYYGMDVWGQGTTVTVSS
D314	Donor 4	IGHV4-59*01	ARGFYGFAGIFEY	13	QVQLQESGPGLVKPSETLSLTCTVSGGSISSYYWSWIRQPPGKGLEWIGYISDSGSTDYNPSLKSRVTISGDTSKNQFSLRLSSVTAADTAVYYCARGFYGFAGIFEYWGQGSLLTVSS
D320	Donor 4	IGHV4-38-2*01	ARTATYYYGVGSDWWLDP	18	QESGPGLVKPSETLSVSCTVSGGSLTSISSSQYWWGWIRQPPGEGLEWIGSIYNDGSTYYNPSLKSRVTLSVDSSKDQFSLELSSVTAADTAVYYCARTATYYYGVGSDWWLDPWGPGTLVTVSS
D321	Donor 4	IGHV4-30-4*01	ARENVGKQQLYTD	13	QVQLQESGPGLVKPSQTLSLTCTVSGHSISSGDYYWSWIRQPPGKGLEWIGNIYSSGNTYYNPSLKSRITISVDTSKKQFSLKLSSVTAADTAVYYCARENVGKQQLYTDWGQGTLVTVSS
D327	Donor 4	IGHV1-69*01	ARDRSSSPWAAYSYGMDV	18	QVQLVQSGAEVKKPGSSVKVSCKASGGTFNTYAFAWVRQAPGQGLEWMGGIIPIFVTPNYAQKFQGRVTITADESTSTVYMELSGLRSEDTAVYYCARDRSSSPWAAYSYGMDVWGQGTTVSVSS
D339	Donor 4	IGHV1-69*01	ARGGGTLWNSVYYFDY	16	QVQLVQSGAEVKKPGSSVKVSCKASGGTFSSFAINWVRQAPGQGLEWMGGIIPIFHTANYAQKFQGRVTITADESTSTAYMELSSLRSEDTAVYYCARGGGTLWNSVYYFDYWGQGSLVTVSS
D340	Donor 4	IGHV4-59*01	ARGSRDFDY	9	QVQLQESGPGLVKPSETLSLTCTVSGGSISSYYWSWIRQPPGKGLEWIGYIYYTGGTNYNPSLKSRVTISVDTSKNQFSLKLSSVTAADTAVYYCARGSRDFDYWGQGTLVTVSS
D342	Donor 4	IGHV3-11*01	ARDPSSSFDY	10	EVQLVESGGGLVKPGGSLRLSCAASGFTFSDYYMSWIRQAPGKGLEWVSNISSRGSAIYYADSVRGRFTISRDNGKNSLSLQMNSLRAEDTAVYYCARDPSSSFDYWGQGTLVTVSS
D346	Donor 4	IGHV3-33*01	ARGVGEADY	9	QVQLVESGGGVVQPGRSLRLSCAASAFTFSTYGMHWVRQAPGKGLEWVALIWYDGSDKYYADSVKGRFTISRDNSKNTLYLQMNSLRVEDTAVYYCARGVGEADYWGQGTLVTVSS
D355	Donor 4	IGHV4-34*01	ARGPLYGLDV	10	QVQLQQWGAGLLKPSETLSLTCAVYGGSFSGYYWSCIRQAPGQGLEWIGEINHTGNTDYNPSLKSRVTMSVDTSKKQFSLNLTSVTAADTAVYYCARGPLYGLDVWGQGTTVIVSS
D358	Donor 4	IGHV1-69*01	ARVEFRSAWHQELGYFDP	18	QVQLEQSGAEVKKPGSSVKVSCKASGGSFSTSGISWVRQAPGQGLEWMGGIIPIFGTANYAQNFQGRVTITADDSTSTAYKELSSLRSEDTAVYYCARVEFRSAWHQELGYFDPWGQGTLVTVSS
D361	Donor 4	IGHV1-18*04	ARHLKSVGEWELFLGFDY	18	QVQLVQSGNEVKKPGASVKVSCKASGYTFSTYGISWVRQAPGQGLEWMGWISAYKGNTKYAQKFQGRVTLTTDTSTTTAYMELTSLRSDDTAVYYCARHLKSVGEWELFLGFDYWGQGTQVTVSS
D364	Donor 4	IGHV4-59*01	VRTYGHYYDSIGYSLGHFDL	20	QVQLQESGPGLVKPSETLSLTCTVSGGSISTFYWSWIRQPPGKGLEWIGYIYYNGNTNYSPSLKSRVTISIDTSKNQFSLKMNSVTAADTAVYFCVRTYGHYYDSIGYSLGHFDLWGRGTLVTVSS
D371	Donor 4	IGHV3-30-3*01	ARVRGYCSGGSCYADY	16	EVQLVESGGGVVQPGRSLRLSCAASGITFSSSAMHWVRQAPGKGLEWVTVISYDGTNKYYADSVKGRFTISRDNSKNTLFLQMNSLRAEDTAMYYCARVRGYCSGGSCYADYWGQGTLVTVSS
D375	Donor 4	IGHV1-69*01	ARGARTHLWSPENNWFDP	18	QVQLVQSGAEVKKPGSSVKVSCKASGGTFTTYTITWVRQAPGQGLEWMGGITPIFGTPNYAQSFQGRVTITADESTNTAYMELSSLRSEDTAVYYCARGARTHLWSPENNWFDPWGQGTLVIVSS
D387	Donor 4	IGHV3-30*03	AKDRQKDFDS	10	RPQLMQSGGGVVQPGTSLRLSCVASGFNLRDYGIHWVRQTPGKGLEWVAVISYDGREKQYADSVKGRFTISRDTSKKTIYLQMTGLRLDDSAIYYCAKDRQKDFDSWGQGSLVTVSS

Supplementary Table 3. Next-generation sequencing (NGS) of antibody heavy chain repertoires of four HEV vaccinated donors. [a]

Donor (N_{Total})	Time point	N_{Raw}	$N_{Assigned}$	<Length> (nt)	N_{Usable} (%)
	Pre	1,857,948	1,598,184	579.5	1,373,588 (85.9%)
	1W1	2,069,386	1,780,121	622.3	1,628,972 (91.5%)
Donor 1	1M1	2,502,139	1,961,638	583.7	1,644,980 (83.9%)
	2W1	1,926,733	1,713,971	618.6	1,557,602 (90.9%)
(14,194,382)	2M1	1,670,534	1,425,651	611.8	1,261,899 (88.5%)
	3W1	1,971,092	1,769,893	611.3	1,609,429 (90.9%)
	3M1	2,196,550	1,859,893	605.2	165,870,8 (89.2%)
	Pre	1,569,670	1,351,103	584.3	1,223,635 (90.6%)
	1W1	1,771,142	1,494,088	584.7	1,394,453 (93.3%)
Donor 2	1M1	1,669,954	1,409,789	585.5	1,310,536 (93.0%)
	2W1	3,016,770	2,474,964	585.4	2,323,737 (93.9%)
(13,559,318)	2M1	2,016,115	1,645,829	585.0	1,509,397 (91.7%)
	3W1	1,947,429	1,631,893	587.9	1,504,034 (92.2%)
	3M1	1,568,238	1,317,931	588.5	1,221,522 (92.7%)
	Pre	1,537,855	1,313,467	576.7	1,177,955 (89.7%)
	1W1	1,865,343	1,520,347	571.0	1,345,562 (88.5%)
Donor 3	1M1	2,143,356	1,699,341	587.7	1,527,478 (89.9%)
	2W1	1,946,351	1,642,733	574.2	1,442,546 (87.8%)
(13,738148)	2M1	2,040,047	1,640,697	586.5	1,489,080 (90.8%)
	3W1	1,945,147	1,666,154	571.5	1,477,457 (88.7%)
	3M1	2,260,049	1,793,767	586.7	1,608,914 (86.7%)
	Pre [b]	2,551,508	2,129,389	569.7	1,988,627 (93.4%)
	1W1	3,096,245	2,182,812	576.8	1,927,060 (88.3%)
Donor 4 [b]	1M1 [b]	2,183,009	1,649,343	564.2	1,466,490 (88.9%)
	2W1	2,276,601	1,713,482	573.4	1,474,998 (86.1%)
(16,948,018)	2M1 [b]	2,068,443	1,557,848	560.3	1,377,416 (88.4%)
	3W1	2,439,948	1,678,417	585.4	1,453,143 (86.6%)
	3M1 [b]	2,332,264	1,786,560	571.8	1,627,854 (91.1%)

[a] Antibody heavy chain libraries were prepared following a previously reported 5'-RACE PCR protocol for unbiased repertoire analysis. Human IgG and IgM reverse primers were used for template preparation, with sequencing performed on the Ion S5 GeneStudio platform using a 530 chip the Ion Personal Genome Machine (PGM) system. Listed items include donor name (total number of barcoded raw reads), time point, number of raw reads for each time point, number of heavy chains with assigned germline genes at a cutoff E-value of 10^{-3}, average read length, and number and percentage ($N_{Usable}/N_{Assigned}\times 100\%$) of usable sequences after *Antibodyomics* pipeline processing and filtering with a cutoff of 250 bp for variable gene alignment.

[b] For four time points of donor 4 vaccination, the mixed heavy chain libraries were sequenced on the Ion Personal Genome Machine (PGM) system using a 318 v2 chip. The PGM datasets were combined with the S5 datasets to increase the sequencing depth and repertoire coverage.

Supplementary Table 4. Expression and functional validation of the reconstituted mAbs with selected heavy chain variants from donor 1 paired with their respective wild-type light chains. [a]

ID	Human IgG	HEV p239(1) binders	Yield [b] (mg/L)	Reactivity (EC_{50} ng/mL) [b]							CDRH3	Amino acid sequence of variable domain
				p239(1)	p239(3)	p239(4)	E2(1)	E2(2)	E2(3)	E2(4)		
A219_3W1_1375961	1.022	3.451	921.6	66.7	114.0	17.9	330.0	687.5	275.4	>1000	ARYSTPDRGHYYYNGLDV	RVQLVHLGAEVKKPGSSMKVSCKASGGTFSDFAINWVRQAPGQGLEWMGAIIPIFGTVNYAQKFQGRVTITADESTNTVYMELSSLRSEDTAVYYCARYSTPDRGHYYYYNGLDVWGQGTTVTVSS
A219_3W1_1048294	2.493	4.222	652.8	>1000	2.7	6.7	12.7	14.8	13.9	279.2	ARYSTPDRGHYYYNGLDV	EVQLVQSGAEVKKPGSSMKVSCKASGGTFSDFAINWVRQAPGQGLEWMGGIIPIFGTVNYAQKFQGRVTITADESTNTVYMELSSLRSEDTAVYYCARYSTPDRGHYYYYNGLDVWGQGTTVTVSS
A225_1M1_9527	2.997	3.168	8.8	878.1	863.6	382.4	760.3	>1000	505.7	>1000	ARGDTVMVKYYYYGMDV	QVQLVQSGAEVKKPGSSVKVSCKASGGTFSSYAISWVRQAPGQGLEWMGGIIPIFGTANYAQKFQGRVTITADESTSTAYMELSSLRSEDTAVYYCARGDTVMVKYYYYYGMDVWGQGTTVTVSS
A225_3W1_1070627	0.016	0.028	-	-	-	-	-	-	-	-	ATRVIQLWLNYYYYGLDV	QMQLVQSGAEVKQLGSSVKVSCKAHGGTSIRYAISWVRQAPGQGLEWVGGIIPVFGTTDYAHRFQGGVTITADDSTSTAYMELSSLRSADHGVYYCATRVIQLWLNYYYYGLDVWGQGTTVTVSS
A225_3W1_962724	2.302	3.366	262	>1000	>1000	>1000	>1000	>1000	>1000	>1000	ATRGDTTMVNYYYYGLDV	EVQLVQSGAEVKQPGSSGKVSCKASGGTVNPYAISWVRQAPGQGLEWVGGIIPVFGTTDYAQRFQGRVTITADDSTSTAYMELSSLRSADTAVYYCATRGDTTMVNYYYYGLDVWGQGTTVTVSS
A258_3W1_1912622	1.319	0.515	302	>1000	>1000	>1000	>1000	>1000	>1000	>1000	ARANIVVAGRHHFHGMDV	VQLVQSGAEVKKPGASVKVSCKASGYTFTTLNGISWVRQAPGQGLEWMGWISAYNGNTNYAQKFQGRVTMTTDTSTMTAYMELRSLRSDDTAVYNCARANIVVAGRHHFHGMDVWGQGTTVTVSS
A258_3W1_105990	1.485	3.622	4.6	1.6	13.5	24.3	7.4	11.3	>1000	>1000	ARANIVVAGRHHFHGMDV	QVQLVQSGAEVKKPGASVKVSCKASGYTFTTYGISWVRQAPGQGLEWMGWISVYNGNTKYAQKFQGRVTMTTDTSTMTAYMELRSLRSDDTAVYNCARANIVVAGRHHFHGMDVWGQGTTVTVSS
A258_3W1_1940081	3.159	3.640	0.84	1.7	33.5	34.6	6.5	9.2	62.0	131.7	ARANIVVAGRHHFHGMDV	QIHAVQSGAEWKKPGASVKVSCKASGYTFTTYGISWVRQAPGQGLEWMGWISAYNGNTNYAQKFQGRVTMTTDTSTMTAYMELRSLRSDDTAVYNCARANIVVAGRHHFHGMDVWGQGTTVTVSS
A275_1M1_1289740	2.777	2.972	0.84	3.7	2.5	2.5	16.0	20.0	13.7	19.9	ARVSGCGGHCYLGAAIDY	RWQLQESGPGLVKPSETLSLTCTVSRDSISSYYWSWIRQPPGKGLEWIGFIYHTGSTNYNPSLKSRVTMSVGTSRDQFSLRLTSVTSADTAVYYCARVSGCGGHCYLGAAIDYWGQGTLVTVSS
A275_2W1_1580262	2.540	3.509	22.26	4.4	7.4	6.7	6.9	6.4	6.4	9.5	ARVSGCGGYCYLGAAIDF	QVQLQESGPGLVKPSETLSLTCTVSRDSISSYYWSWIRQPPGKGLEWIGFIYHTGSTDYNPSLKSRVTMSVDTSRDQFSLRLTSVTSADTAVYYCARVSGCGGYCYLGAAIDFWGQGTLVTVSS
A275_3M1_1028378	3.444	3.474	3.2	0.7	0.6	1.5	2.1	1.8	2.0	1.3	ARVSGCGGYCYLGAAIDY	QVQLQESGPGLVKPSETLSLTCTVSRDSISSYYWSWIRQPPGKGLQWIGFIYHSGSTIYNPSLKSPVTMSIDTSRDQFSLRLTSVTSADTAVYYCARVSGCGGYCYLGAAIDYWGQGTLVTVSS
A275_3W1_1723224	2.786	3.039	1.54	2.2	1.8	1.8	1.3	1.6	0.6	1.2	ARVSGCGGYCYLGAAIDY	RVQLQESGPGLVKPSETLSLTCNVSRDSISSHYWSWIRQPPGKGLQWIGFIYHTGSTSYNPSLKSRVTMSLDTSRDQFSLRLTSVTSADTAVYYCARVSGCGGYCYLGAAIDYWGQGTLVTVSS
A275_3M1_353239	3.287	3.681	3.2	10.7	10.0	9.5	10.8	10.3	6.8	10.2	ARVSGCGGYCYLGAAIDY	QVQLQESGPGLGEPSETLSLTCTVSRDSISSYYWSWIRQPPGKGLAWIGFIYHSGSTIYNPSLKSRVTMSIDTSRDQFSLRLTSVTSADTAVYYCARVSGCGGYCYLGAAIDYWGQGTLVTVSS

A275_3W1_160695	3.617	3.729	10.4	1.3	1.3	1.4	2.7	2.5	2.8	1.5	CARVSGCGGHCYLGAAIDY	QVQLQESGPGLVKPSETLSLTCTVSRDSIYSYYWTWIRQPPGKGLEWIGFLSHTGSTNYNPSLKSRISTMSVDASRDQFSLRLTSVTSADTAVYYCARVSGCGGHCYLGAAIDYWGQGTLVTVSS
A275_2W1_1491742	3.402	0.011	-	-	-	-	-	-	-	-	ARVSAAVVTGYLGAAIDH	PVQLQVSGPGLVKPSETLSLTCSVSRGSISSYYWSWIRQAPGKGLEWIGFYSYHTGSTNYNPSLESRVTMSVDTSRDQFSLRLTSVTSADTAVYYCARVSAAVVTGYLGAAIDHWGQGTLVTVSS
A275_2W1_1817025	0.044	0.008	-	-	-	-	-	-	-	-	ARVSGCGGYCYLGAAIDY	VQLGQESGPGLVKPSETLSLTCSVSGDSISSYYWSWIRQPPGTGLGNGIGFSYHTGRANYNPSLESRVTMSVDTSRDQFSLRLTSVTSADTAVYYCARVSGCGGYCYLGAAIDYWGQGTLVTVSS
A275_2W1_767505	0.456	0.021	-	-	-	-	-	-	-	-	APSVWVAVVTAILVPRFDY	RVQLQESGPGLVKASETLSLTCSVSGDSISSYYWSWIRQPPGKGLEWIGFIYHTGSTKYNPSLESRVTMSVDTSRSQFSLRLTSVTSADTAVYYCAPSVWVAVVTAILVPRFDYWGQGTLVTVSS
A275_2W1_1890722	0.057	0.072	-	-	-	-	-	-	-	-	ARVSGCGGYCYLGAAIDY	QVQLQESGPGLVKPSETLSLTCSVSRDSHSSYYWSWIRQPPGKGLGNGIGFIYHTGSTNYNPSLSTSRVTMSVDTSRDQFSLRLTSVTSADTAVYYCARVSGCGGYCYLGAAIDYWGQGTLVTVSS
A275_2W1_739441	0.533	0.069	-	-	-	-	-	-	-	-	ARVVGLRWLLGYLGAAIDY	TVQLQESGPGLVKPSETLSLTCSVSRDAISSYYWSWIRQPPGKGLEWIGFIYHTGSTNYNPSLKSRVTMLVDTSRIQFSLRLTSVTSADTAVYYCARVVGLRWLLGYLGAAIDYWGQGTLVTVSS
A275_3W1_1238724	2.504	2.798	2.4	1.6	1.5	1.8	2.0	2.7	2.4	2.4	ARVSGCGGHCYLGAAIDY	QVQLQESGPGLVKPSETLSLTCTVSRDSIYSYYWTWIRQPPGKGLEWIGFLSHTGSTNYNPSLKSRLTMSVDTSRDQFSLRLTSVTSADTAVYYCARVSGCGGHCYLGAAIDYWGQGTLVTVSS
A275_3W1_1355810	0.219	0.203	4.48	2.5	3.5	3.5	3.1	5.3	4.3	4.3	YCARVSGCGGYCYLGAAIDY	QVQLQESGPGLVKPSETLSLTCTVSRDSISTYYWTWIRQPPGKGLEWIGFIYHTGSTNYNPSLKSRGSTQLSLDTSRDQFSLRLTSVTSADTAVYYCARVSGCGGYCYLGAAIDYWGQGTLVTVSS
A275_3W1_1313139	2.662	3.190	3.5	3.0	3.0	3.0	6.4	6.1	4.1	6.8	ARVSGCGGYCYLGAAIDH	QVQLQESGPGLVKPSETLSLTCSVSRESISTHYWTWIRQPPGKGLEWIGFIYHTGSTNYNPSLKSRVTMSIDTSRDQFSLRLTSVTSADTAVYYCARVSGCGGYCYLGAAIDHWGQGTLVTVSS
A275_3W1_1000786	0.904	1.200	14	138.3	79.9	105.6	103.7	213.4	93.4	169.0	ARVSACGGHCYLGAAIDY	PVQLQESAPGLVKPSETLSLTCTVSRDSIYSYYWSWIRQPPGKGLEWIGFIHHTGSTNYNPSLKSRLTMSVDTSRDQFSLRLTSVTSTDTAVYYCARVSACGGHCYLGAAIDYWGQGTLVTVSS
A275_3W1_1038435	0.312	0.067	-	-	-	-	-	-	-	-	ARVSGLLVVTAILVPRFDS	TVQLQESGPGLVKPSETLSLTCTVSRDSISSYYWSWIRQPPGKGLEWIGFIYHTGSTNYNPSLKSRVTMSVDTSRIQFSLRLTSVTSADTAVYYCARVSGLLVVTAILVPRFDSWGQGTLVTVSS
A278_3W1_1207860	3.426	3.431	28.8	3.3	2.5	6.4	29.5	11.1	32.8	16.8	AKEGGSAWNDHAFDI	QVQLVQSGPEVKKPGSSVKVSCKASGGTFNGNAISWVRQAPGQGLEWMGGIIPIFATPNYAQNFQGRVTITADESTSTSYMELTSLISEDTAVYYCAKEGGSAWNDHAFDIWGLGTLVTVSS
A278_2W1_1158676	1.736	0.034	-	-	-	-	-	-	-	-	ATEGGSAWKTMILDI	QVQLVQSGAEVKKPGSSVKVSCKASGGTFNSNAISWVRQAPGQGLEWMGGIIPIFVTANYAQKFQGRVTITADESTSTAYMELSSLRSEDTAVYYCATEGGSAWKTMILDIWGQGTMVTVSS
A278_2W1_1798664	0.225	0.037	-	-	-	-	-	-	-	-	ATEGGSAWNDHAFDI	QVQLVQSGAEVEKPGSSVKVSCKASGGTFNSNAIRWVRQAPGPGLEWMGGIIPIFVTANYAQKFQGRVTITADESTSTAYMELSSLRSEDTAVYYCATEGGSAWNDHAFDIWGQGTMVTVSS
A278_3W1_1043	2.695	3.585	68	7.1	10.2	3.8	9.9	~	7.1	29.8	AAEGGSAWNDHGFDI	QLVQSGAEVKKHRGSSVKVSCKASVGGTFNGNAISWVRQAPGQGLEWMGGIIPIFVTPNYAQKFQGRVTITADESTNTVYMELNSLRSEDTAVYYCAAEGGSAWNDHGFDIWGQGTMVTVSS

A278_3W1_157403	2.913	4.364	8.4	6.0	8.4	3.8	54.9	179.7	11.0	28.8	ASEGGSAWNDHAFDM	QVQLVQSGAEVKKPGSSVKVSCKASGGTFNSNAISWVRQAPGQGLEWMGGIIPIFATPNYAQKFQGRVTITADESTSTVYMELSRLRSEDTAVYYCASEGGSAWNDHAFDMWGQGTMVTVSP
A278_3W1_1198414	3.348	0.569	147.2	371.1	22.8	279.5	>1000	>1000	>1000	>1000	ATEGGSAWNDHGFDI	VQLVQSGAEVKKPGSSVKVSCKASGGTFNGQSAISWVRQAPGQGLEWMGGIIPIFVTPNYAQKFQGRVTITADESTNTVYMELNSLRSEDTAVYYCATEGGSAWNDHGFDIWGQGTMVTVSS
A278_3W1_539907	2.635	4.376	57.6	>1000	>1000	>1000	>1000	>1000	>1000	>1000	AKEGGSAWNDHAFDI	QVRLVQSGPEVKKPGSSVKVSCKASGGTFNGNAISWVRQAPGQGLEWMGGIIPIFATPNYAQNFQGRVTITADESTSTSYMELTSLISEDTAVYYCAKEGGSAWNDHAFDIWGLGTLVTVSS
A283_2W1_1837029	3.273	3.947	0.55	6.3	6.2	5.7	5.8	5.5	3.9	6.0	ARDVVDIVATGWGGVYYFYYGTDV	QLQLQESGPGLVKPSETVSLTCTVSGGSISSSSYHWGWIRQPPGRGLEWVGGIYYSGSTYYNPSLRSRVTISVDTSKKQFSLKLSSVTAADTAVYYCARDVVDIVATGWGGVYYFYYGTDVWGQGTTVTVSS
A283_3W1_1002504	3.001	3.353	1.52	1.5	1.7	2.0	6.5	9.4	6.4	8.2	AIDVVDTVATGGGNYYFYYGTDV	QMQLQESGPGLVKPSETLSLSCTVSGGSISSSSYYWGWIRQPPGKGLEWIGNIYYSGSTYYNPSLKSRVTISVDTSKNQFSLRLNSVTAADTAVYYCAIDVVDTVATGGGNYYFYYGTDVWGQGTTVTVSS
A283_2W1_117337	0.086	0.011	-	-	-	-	-	-	-	-	ARDVVDIVATGGGSYYFYYGIDV	ELQLQESGPGLVKPSETLSLTCTVSGGSISSSSYYWGWIRQPPGKGLEWIGSIYYSGSTHHNPSLRSRVTISVDTSKKQCSLKLSSVTAADTAVYYCARDVVDIVATGGGSYYFYYGIDVWGQGTTVTVSS
A283_2W1_203183	2.886	3.910	873.6	4.2	2.9	3.3	5.8	7.1	5.1	7.2	ARDVVDIVATGGGNFHFYYGMDV	QLQLQEVGPGLVKPSETLSLTCTVSGGSISSSSYYWGWIRQPPGKGLEWIGSIYYSGSTYYNPSLKSRVTISVDTSKNQFSLKLSSVTAADTAVYYCARDVVDIVATGGGNFHFYYGMDVWGQGTTVTVSS
A283_3W1_1822006	0.041	0.017	-	-	-	-	-	-	-	-	ARDAVDTVATGGGNYYFYYGTDV	QLQLQESGPGLVRPSETLSLTCTVSGGSISSSTYYWAWIRQPPGKGLEWIGSVYYSGSAYYNPSLRSRVAISLDTSKNQFSLKLSSGTAADTAVYYCARDAVDTVATGGGNYYFYYGTDVWGHGTTVTVSS
A283_3W1_1968193	0.054	0.015	-	-	-	-	-	-	-	-	ARDVVDVVPTGGGDYYFYYGTDV	ELQLQEVRPGLVRPSETLSLTCTVSGGSVSSSTYYWAWIRQPPGKGLEWIGNIYYSGSAYYNPSLRSRVAISLDTSKNQFSLKLSSVTAADTAVYHCARDVVDVVPTGGGDYYFYYGTDVWGHGTTVTVSS
A283_2W1_566151	2.300	3.643	52.8	10.0	11.0	11.7	13.0	13.5	12.7	33.7	ARDVVDIVATGGGSYYFYYGIDV	TLQLQEYGPGLVKPSETLVLTCTVSGGSISSSSYYWGWIRQPPGKGLEWIGSIYYSGSTHHNPSLRSRVTISVDTSKKQCSLKLSSVTAADTAVYYCARDVVDIVATGGGSYYFYYGIDVWGQGTTVTVSS
A283_3W1_1957753	0.122	0.086	-	-	-	-	-	-	-	-	ARDVVDVVPTGGGDYYFYYGTDV	LQLQVLPGPGLVRPSETLSLTCTVSGGSVSSSTYYWAWIRPAPGKGLEWIGNIYYSGNAYYNPSLRSRVAISLDTSKNQFSLKLSSVTAADTAVYHCARDVVDVVPTGGGDYYFYYGTDVWGHGTTVTVSS

[a] For heavy chain variants, the nomenclature is defined as [Parental antibody]_[Time point]_[Sequence index].
[b] Only HEV p239(1)-specific antibodies were analyzed for protein yield and reactivity to different genotypes HEV capsid proteins.

Supplementary Table 5. Expression and functional validation of the reconstituted mAbs with selected heavy chain variants from donor 2 paired with their respective wild-type light chains. [a]

ID	Human IgG	HEV p239(1) binders	Yield [b] (mg/L)	Reactivity (EC_{50} ng/mL) [b]							CDRH3	Amino acid sequence of variable domain
				p239(1)	p239(3)	p239(4)	E2(1)	E2(2)	E2(3)	E2(4)		
B128_2M1_298576	0.044	0.013	-	-	-	-	-	-	-	-	ANHDPDYYYYAMDV	VQAWVQSGAEVKKPGSSVKVSCKASGGTFTNYAISWVRQGPGQGLEWMGGIIPVFGTPNYAQNFQGRATITADESTSTAYMELSSLRSGDTAVYYCANHDPDYYYYAMDVWGQGTTVTVSS
B128_2W1_1500226	2.679	0.520	21.6	7.4	7.3	7.1	53.8	35.6	23.8	126.6	ANHYSDFYYYAMDV	QWQLVQSGAEVKKPGSSVKVSCKASGGTFSNYAISWVRRAPGQGLEWMGGIIPIFGIPNYAQKFQGRVTLTADESTSTAYMELSSLRSEDTAIYYCANHYSDFYYYAMDVWGQGTTVTVSS
B128_2W1_2280015	0.516	0.068	-	-	-	-	-	-	-	-	ANHDSDYYYYGMDV	QVHVVDAGAEVKKPGAIVKVSCKASGGTSGNYAISWVRQAPGQGLEWMGGIIPIFGRPNYAQKFQGRVTITADESTSTAYMELSSLRSDDTAVYYCANHDSDYYYYGMDVWGQGTTVTVSS
B128_2W1_2558281	0.883	0.011	-	-	-	-	-	-	-	-	ANHDSDYYYHRMDV	QVELVQSGAEVKKPGSSVKVSCKASGGTFSNYAISWVRQAPGSKGLEWMGGIIPIFGITENYAQKFQGRVTITADESTSTAYMELSSLRSEDTAVYYCANHDSDYYYHRMDVWGQGTTVTVSS
B128_2W1_373609	1.703	0.007	-	-	-	-	-	-	-	-	ANHDSDYYFYVMDV	QWQLVQSWAEVKKPGSSVKVSCKASGGTFRNYAISWVRQAPGQGLEWMGGIIPIFGTPNYAQKFQGRVTITADESTSTAYMELSSLRSEDTAVYYCANHDSDYYFYVMDVWGQGTTVTVSS
B137_1M1_1023324	1.414	3.499	75.2	>1000	>1000	>1000	>1000	>1000	>1000	>1000	ARDAPWRSSSQITNYFDY	QVQLVESGGGVVQPGRSLRLSCAASGFTFSNYAMRWVRQAPGRGLEWVAVISYDGSNKYYVDSVKGRFTISRDNSKNTLYLQMNSLRAEDTAVYYCARDAPWRSSSQITNYFDYWGQGTLVTVSS
B137_1M1_1431597	0.211	0.050	-	-	-	-	-	-	-	-	ARDAPWRSSSQITNYFDY	PVELGRGEALDAQPGRLLRLSCAASGFTFSYYAMRWVRQTPGKGLGWVAVISYDGSNEYYVDSVKGRFTISRDNSKNTLYLQMNSLRAEDTAVYYCARDAPWRSSSQITNYFDYWGQGTLVTVSS
B137_1M1_582382	0.220	0.027	-	-	-	-	-	-	-	-	ARDAPWRSSSQITNYFDY	VQLVIWGEGVVQPGRSLRLLPCAASGFTFSYYAMRWVRQAPGKGLGWVAVISYDGSNEYYVDSMKGRFTISRDNSKNTLYLQMNSLRAEDTAVYYCARDAPWRSSSQITNYFDYWGQGTLVTVSS
B137_2M1_435677	0.827	0.385	28	105.8	465.7	256.1	442.5	>1000	150.6	472.4	ARDAPWRSSSQITNYFDY	QVQLVESGGGVVQPGRSLRLSCAASGFTFSSYAMRWVRQAPGKGLEWVAVISYDVSNKYYVDSVKGRFTISRDNSKNTLYLQMNSLRAEDTAVYYCARDAPWRSSSQITNYFDYWGQGTLVTVSS
B137_2W1_1009231	4.037	3.515	9.6	>1000	>1000	>1000	>1000	>1000	867.1	>1000	ARDAPWRSSSQITNYFDY	QVQLVESGGGVVQPGRSLRLSCAASGFTFSSYAMRWVRQAPGKGLEWVAVISYDVSNKYYVDSVKGRFTISRDNSKNTLYLQMNSLRAEDTAVYYCARDAPWRSSSQITNYFDYWGQGTLVTVSS
B137_2W1_2716440	0.651	0.010	-	-	-	-	-	-	-	-	ARDAPWRSSSQITNYFDY	VQLVEALGGGVVQPGRSLRLSCAASGFTFSSYAMRWVRQAPGKGLEWVAVISYDVSNKYYVDSVKGRFTNSRDNSKNTLYLQMNSLRAEDTAVYYCARDAPWRSSSQITNYFDYWGQGTLVTVSS
B137_2W1_3498	0.240	0.013	-	-	-	-	-	-	-	-	ARDAPWRSSSQITNYFDY	QVQLVESWGGVVHPGRSLRLSCAASGFTFSSYAMRWVRQAPGKGLEWVAVISYDVSNKYYVDSSEGRFTISRDNSKNTLYLQMNSLRAEDTAVYYCARDAPWRSSSQITNYFDYWGQGTLVTVSS
B137_3W1_1500080	0.528	0.084	-	-	-	-	-	-	-	-	VRDVPWRSSSQITNYFDN	VQLGWESGGGVVQPGRSLRLSCAASGFTFSYYAMQWVRQAPGKGLEWVAVISYDGSNEYYVDSVKGRFTISRDNSKNTLYLQMNSLRAEDTAVYYCVRDVPWRSSSQITNYFDNWGQGTLVIVSS
B137_3W1_1867950	0.328	0.033	-	-	-	-	-	-	-	-	VRDVPWRSSSQITNYFDN	QLVESVGGGVGAQPGRSLRLSCAASGFTFSYYAMQWVRQAPGKGLEWVAVISYDGSNEYYVDSV

												KGRFTISRDNSKNTLYLQMNSLRAEDTAVYYCV<br>RDVPWRSSSQITNYFDNWGQGTLVIVSS
B137_3W1_628767	3.837	3.699	12.8	154.3	814.3	282.9	281.6	163.0	823.8	>1000	VRDVPWRSSSQ<br>ITNYFDN	RVQLVESGGGVVQLGRSLRLSCAASGFTFSTYA<br>MQWVRQAPGKGLEWVAVISYDGSNEYYVDSV<br>KGRFTISRDNSKNTLYLQMNSLRAEDTAVYYCV<br>RDVPWRSSSQITNYFDNWGQGTLVIVSS
B137_3W1_882686	0.234	0.027	-	-	-	-	-	-	-	-	VRDVPWRSSSQ<br>ITNYFDN	SWLVESGGGAGVPAGRSLRLSCAASGFTSVTYA<br>MQWVRQAPGKGLEWVAVISYAGSNEYYVVSV<br>KGRLPISRDNSKNTLYLQMNSLRAEDTAVYYCV<br>RDVPWRSSSQITNYFDNWGQGTLVIVSS
B137-germline	1.150	3.615	15.2	>1000	>1000	>1000	>1000	>1000	>1000	>1000	ARDVPWRSSSH<br>ITNYFDY	QVQLVESGGGVVQPGRSLRLSCAASGFTFSSYA<br>MHWVRQAPGKGLEWVAVISYDGSNKYYADSV<br>KGRFTISRDNSKNTLYLQMNSLRAEDTAVYYCA<br>RDVPWRSSSHITNYFDYWGQGTLVTVSS
B170_2W1_1035372	0.043	0.010	-	-	-	-	-	-	-	-	ARGGTGNPWR<br>DAYYYGMDV	QVQLVQFGAEVKKPGSSVKVSCKASGGTFSSYA<br>INWVRQPRVQGMRWMGRIIPNLATATRYQKFE<br>GRVTITADESTSTAYMELSSLRSEDTAVYYCAR<br>GGTGNPWRDAYYYGMDVWGQGTTVTVSS
B170_2W1_1210881	2.897	0.011	-	-	-	-	-	-	-	-	ARGGTGNPWR<br>GAYYYGMDV	QVQLVQFGAEVKKPGSSVKVSCKASGGTFSSYA<br>INWVRQAPGQGLEWMGGIIPIFATANHAQKFQG<br>RVTITADESTSTAYMELSSLRSEDTAVYYCARG<br>GTGNPWRGAYYYGMDVWGKGTTVTVSS
B170_2W1_2405774	0.080	0.016	-	-	-	-	-	-	-	-	ARGGTGTPWR<br>DAYYYGMDV	QVQLVQSGAEVKKPGSSVKVSCKASGAFSSYAI<br>SWLRQAPGQGLDGLGGIIPIFATSNYAQKFQGR<br>VTITADESTSTAYMELSSLRSEDTAVYYCARGG<br>TGTPWRDAYYYGMDVWGQGTTVTVSS
B170_2W1_1232631	3.446	1.594	9.5	>1000	30.0	74.8	>1000	>1000	>1000	>1000	ARGGTGNPWR<br>DAYYYGMDV	QVQLVQSGAEVKKPGSSVKVSCKASGGTFSSYA<br>INWVRQAPGQGLEWMGGIIPIFATANHAQKFQG<br>RVTITADESTSTAYMELSSLRSEDTAVYYCARG<br>GTGNPWRDAYYYGMDVWGQGTTVTVSS
B170_3W1_1172416	0.043	0.018	-	-	-	-	-	-	-	-	ARGGTGNPWR<br>DAYYFGMDV	VQLVHLGAEVKKPGSSVVRVSCKASGVTFSTSA<br>ISWVRQAPGQGLESMGGIIPIFATAHYAQNFQGR<br>VTITADESTSTAYMELNSLRSEDTALYYCARGG<br>TGNPWRDAYYFGMDVWGQGTTVTVSS
B170_3W1_166243	3.259	3.217	137.6	2.4	1.3	3.5	951.4	3.2	485.4	5.0	ARGGTGNPWR<br>DAYYYGMDV	EVHLVQSGAEVRKPGSSVKVSCKASGGTFNTY<br>AISWVRQAPGQGLEWMGGIIPIFATPNHAQKFQ<br>DRVTITADESTSTAYMELSSLTSEDTAVYYCAR<br>GGTGNPWRDAYYYGMDVWGQGTTVTVSS
B170_3W1_1206743	1.970	3.576	61.2	16.8	9.2	13.1	20.2	18.9	18.3	9.6	AKGGTGNPWR<br>DAHYYLMDV	RVQLVQSGAEVKKPGSSVKVSCKASGGTFSTYA<br>INWVRQAPGQGLEWMGGIIPIFATANHAQQFQG<br>RVTITADESTSTAYMELSSLTSEDTAVYYCAKG<br>GTGNPWRDAHYYLMDVWGQGTTVAVSS
B170_3W1_1400703	0.158	0.141	4	129.2	191.4	181.3	268.1	306.2	158.2	270.4	ARGGTGNPWR<br>DAYYYGMDV	PVQLVQSGAEVKKPGSSAKVSCKASGGAFSTYA<br>ISWVRQAPGQGLEWMGGIIPIFATASYAQKFQG<br>RVTITADESTSTAYMELNSLRSEDTALYYCARG<br>GTGNPWRDAYYYGMDVWGQGPTVTVSS
B170_3W1_153102	3.240	1.772	5.4	>1000	11.9	59.2	824.5	>1000	142.5	>1000	ARGGTGNPWT<br>DGYYYGMDV	QVQLVQSGAEVKKPGSSVKVSCKASGGTFSTY<br>AISWVRQAPGQGLEWMGGIIPIFGTANYAQKFQ<br>GRVTITADESTSTANMELSSLRSEDTAVYYCAR<br>GGTGNPWTDGYYYGMDVWGQGTTVTVSS

[a] For heavy chain variants, the nomenclature is defined as [Parental antibody]_[Time point]_[Sequence index].
[b] Only HEV p239(1)-specific antibodies were analyzed for protein yield and reactivity to different genotypes HEV capsid proteins.

Supplementary Table 6. Expression and functional validation of the reconstituted mAbs with selected heavy chain variants from donor 3 paired with their respective wild-type light chains. [a]

ID	Human IgG	HEV p239(1) binders	Yield [b] (mg/L)	Reactivity (EC_{50} ng/mL) [b]							CDRH3	Amino acid sequence of variable domain
				p239(1)	p239(3)	p239(4)	E2(1)	E2(2)	E2(3)	E2(4)		
C144_2W1_1224313	0.019	0.011	-	-	-	-	-	-	-	-	ARDLRSSSSWYSNYYMDV	QVQLVQSGAEVMKPGSSVKVSCKASGGTFSTYAISWVRQAPGQGLEWMGGIIPIFGAANYAQKFQGRVTITADESTSTAYMELSSLRSEDTAVYYCARDLRSSSSWYSNYYMDVWGKGTTVTVSS
C144_2W1_1489111	0.220	0.009	-	-	-	-	-	-	-	-	ARDLRSSSSWYSNYYMDV	EVQLVQSGAEVKKPGSSVKVSCKASGGTFSTYAISWVRQAPGQGLEWMGGIIPIFGAANYAQKFQGRVTITADESTSTAYMELSSLRSEDTAVYYCARDLRSSSSWYSNYYMDVWGKGTTVTVSS
C144_2W1_744852	0.040	0.006	-	-	-	-	-	-	-	-	ARDLRSSSSWYSNYYMDV	QVQLVRSGATVRKPGSSVKVSCKASGGTFSTYAISWVRAAPGQGLEWMGGKSIPIFGAANLTAQKFQGRVTITADESTSRAYMELSSLRSEDTAVYYCARDLRSSSSWYSNYYMDVWAKGTTGTVSS
C144_2W1_707129	2.973	3.869	4.24	11.8	9.9	9.6	6.6	18.5	9.2	8.3	ARDLRSSSSWYSNYYMDV	QVQLVQSGAEVKKPGSSVKVSCKASGGTFSTYAISWVRQAPGQGLEWMGGIIPIFGAANYAQKFQGRVTITADESTSTAYMELSSLRSEDTAVYYCARDLRSSSSWYSNYYMDVWGKGTTVTVSS
C172_2M1_1786057	2.272	2.815	4.8	4.4	5.7	19.9	22.5	10.8	24.8	20.5	ARETTVTPDPRYYYYYYMDV	VQLVQSGAEVKKPGASVKVSCKASLGYYLTSYGISWVRQAPGQGLEWMGWISGYNGNKNFAQKFQGRITMTTDTSTSTAYMELRSLRSDDTAVYYCARETTVTPDPRYYYYYYMDVWGKGTTVTVSS
C172_2M1_849975	3.300	3.341	1180.8	43.2	>1000	>1000	>1000	371.5	>1000	>1000	ARETTVTPDPRYYYYYYMDV	QVQLVQSGAEVKKPGASVKVSCKASGYTFTSYGISWVRQVPGQGLEWMGWISGSNGNKNFAQKFQGRITMTTDTSTSTAYMELRSLRSDDTAVYYCARETTVTPDPRYYYYYYMDVWGKGTTVTVSS
C172_2M1_1681057	0.447	0.023	-	-	-	-	-	-	-	-	ARETTVTPDPRYYYYYYYMDV	QIQLVQSGAEVEKPGGLVRYSCKASGYTFTSYGISWVRQAPGQGLEWMGWIDGYNGNKNFAQKFQDRITMTTDTSTSTAYMELRSLRSDDTAVYYCARETTVTPDPRYYYYYYYMDVWGKGTTVTVSS
C172_2M1_1129213	1.539	3.381	1.68	51.7	49.7	45.7	25.8	44.3	63.4	115.3	ARETTVTPDPRYYYYYYMDV	QVQLVQSGAEVKKPGASVKVSCKASGYTFTSYGISWVRQAPGQGLEWMGWISGYNGNKNFAQKFQGRITMTTDTSTSTAYMELRSLRSDDTAVYYCARETTVTPDPRYYYYYYMDVWGKGTTVTVSS
C172_3W1_1750726	0.061	0.010	-	-	-	-	-	-	-	-	ARETTMTPDPRYNYYYMDV	QVQLVQSGGEVKKPGASVKVSCRASGYTFTLDGVNWGAQAPGQGLEWMGWISGYNGNTNYAQNFQGRVTLTTDTSTTTAYMELRSLRSDDTAVYYCARETTMTPDPRYYNYYYMDVWGTGTTVTVSS
C172_3W1_1877615	0.110	0.021	-	-	-	-	-	-	-	-	ARETTMTPDPQYYNYYYMDV	RVELVQSGGGERRPGASVEVSCEASGYTFTSYGISWARQAPGQGLEWMGWISGYNGNKNYAQNFQGRVTSTTDTSTTTAYMELRSLRSDDTAVYYCARETTMTPDPQYYNYYYMDVWGTGTTVTVSS
C172_3W1_1027609	1.480	4.169	5.4	24.5	23.0	20.3	14.5	39.0	30.9	42.7	ARETTMTPDPRYNYYYIDV	QVQLVQSGGEVKKPGASVKVSCRASGYTFTAYGVNWVRQAPGQGLEWMGWISGYNGNKNYAQNFQGRVTLTTDTSTTTAYMELRSLRSDDT

												AVYYCARETTMTPDPRYYNYYYIDVWGTGT TVTVSS
C172_3W1_1071920	2.272	1.318	758.4	>1000	>1000	>1000	>1000	>1000	>1000	>1000	ARETTMTPRPPRY YNYYYMDV	QVQLVQSGGEVKKPGASVKVSCRASGYTFTA YGVNWVRQAPGQGLEWMGWISGYNGNTNY AQNFQGRVTLTTDTSTTTAYMELRSLRSDDT AVYYCARETTMTPRPPRYYNYYYMDVWGTG TTVTVSS
C172_3W1_1339916	1.675	3.561	21	898.1	966.8	>1000	>1000	>1000	>1000	>1000	ARETTMTPDPRY YNYYYMDV	QVQLVQSGGEVKKPGASVKVSCRASGYTFTA YGVNWVRQAPGQGLEWMGWISGYNGNTNY AQNFQGRVTLTTDTSTTTAYMELRSLRSDDT AVYYCARETTMTPDPRYYNYYYMDVWGTGT TVTVSS
C183_3W1_1018432	0.321	0.014	-	-	-	-	-	-	-	-	ARERGGSIVVPAA LYMDV	VQLVQSGAEVKKPGASVKVSCKASGYTIRYS YGIHWVRHGPGQRLEWMGRITTGNGNTKYS QKLQDRITITRDTSASTAYMELSSLRSEDTAV YYCARERGGSIVVPAALYMDVWGKGTTVTV SS
C183_3W1_1094562	3.614	3.635	1176	2.3	1.4	8.3	7.8	2.1	11.2	11.9	ARERGGSIVVPAA LYMDV	QVQLVQSGAEVKKPGASVKVSCKASGYTFTR YGIHWVRQAPGQRLEWMGRITTGNGNTKYS QKLQDRITITRDTSASTAYMELSSLRSEDTAV YYCARERGGSIVVPAALYMDVWGKGTTVTV SS
C183_3W1_1905798	2.681	0.027	-	-	-	-	-	-	-	-	ARERGGSIVVPAA PYMDV	EVQLVQSGAEVKKPGASVKVSCKASGYTFTR YGIHWVRQAPGQRLEWMGRITTGNGNTKYS QKLQGRITITRDTSASTAYMELSSLRSEDTAV YYCARERGGSIVVPAAPYMDVWGKGTTVTV SS

[a] For heavy chain variants, the nomenclature is defined as [Parental antibody]_[Time point]_[Sequence index].

[b] Only HEV p239(1)-specific antibodies were analyzed for protein yield and reactivity to different genotypes HEV capsid proteins.

Supplementary Table 7. Expression and functional validation of the reconstituted mAbs with selected heavy chain variants from donor 4 paired with their respective wild-type light chains. [a]

ID	Human IgG	HEV p239(1) binders	Yield (mg/L)	Reactivity (EC50 ng/mL)							HCDR3	Amino acid sequence of variable domain
				p239(1)	p239(3)	p239(4)	E2(1)	E2(2)	E2(3)	E2(4)		
D321_1W1_691142	1.991	2.842	8.8	255.3	208.4	112.6	250.7	305.7	260.3	>1000	AREDVGKQQL<br>YTD	QVQLQESGPGLVKPSQTLSLTCTVSGGSISSGD<br>YYWSWIRQPPGKGLEWIGLIYYSGSTYYTPSL<br>KSRVTISVDTSKNQYSLKLSSVTAADTAVYYC<br>AREDVGKQQLYTDWGQGTLVTVSS
D321_2W1_128207	0.263	0.006	-	-	-	-	-	-	-	-	AREDVGKQQL<br>YTD	RVQLRESGPGLVKPSQTLSLTCTVSGGSISSGD<br>YYWSWIRQPPGKGLEWIGLIYYSGSTYYNPSL<br>KSRVTISVDTSKNQYSLKLSSVTAADTAVYYC<br>AREDVGKQQLYTDWGQGTLVTVSS
D321_2W1_1532649	0.233	0.015	-	-	-	-	-	-	-	-	AREDVGKQQL<br>YTD	RVQLEESGPGLVKPSQTLSLTCTVSGGSISSGD<br>YYWSWIRQPPGRGLEWIGLIYYSGSTYYNPSL<br>KSRVTISVDTSKNQYSLKLRSVTAADTAVYYC<br>AREDVGKQQLYTDWGQGTLVTVSS
D321_2W1_666957	0.297	0.013	-	-	-	-	-	-	-	-	AREDVGKQQL<br>YTD	QVQLQESGPGLVKPSQTLSLTCTVSGGSISSGD<br>YYWSWIRQPPGKGLEWIGLIHYRGSTYYNPSL<br>KSRVTISVDTSKNQYSLKLSSVTAADTAVYYC<br>AREDVGKQQLYTDWGQGTLVTVSS
D321_2W1_1165156	2.703	3.614	4	189.7	162.7	172.9	211.1	281.1	196.3	350.5	AREDVGKQQL<br>YTD	QVQLQESGPGLVKPSQTLSLTCTVSGGSISSGD<br>YYWSWIRQPPGKGLEWIGLIYYSGSTYYNPSL<br>KSRVTISVDTSKNQYSLKLSSVTAADTAVYYC<br>AREDVGKQQLYTDWGQGTLVTVSS
D321_2W1_2010290	3.236	4.012	6.6	46.4	77.2	37.3	74.7	42.0	64.9	160.9	AREDVGKQQL<br>YTD	VQLQESAPGLRLKTLTTLSLTCTVSGGSISSGD<br>YYWSWIRQPPGKGLEWIGLIYYTGSTYYNPSL<br>KSRVTISVDTSKNQYSLKLSSVTAADTAVYYC<br>AREDVGKQQLYTDWGQGTLVTVSS
D321_2W1_2044950	0.708	0.028	-	-	-	-	-	-	-	-	AREDVGKQQL<br>YTD	QLQESGPGLVKPSQTLSLTCTVSGGSISSGDYL<br>EAWSWIRQPPGKGLEWIGLIYYSGSTYHNPSL<br>KSRVTISVDTSKNQYSLKLSSVTAADTAVYYC<br>AREDVGKQQLYTDWGQGTLVTVSS
D339_2W1_1079175	2.110	0.017	-	-	-	-	-	-	-	-	ARGGGDVLGIR<br>FYYFDY	QVQLVQSGAEVKKPGSSVKVSCKASGGTFSSY<br>AISWVRQAPGQGLEWMGGIIPIFLTANNAQKL<br>QGRVTITADESTSTAYMELSSLRSEDTAVYYC<br>ARGGGDVLGIRFYYFDYWGQGTLVTVSS
D339_2W1_640193	3.629	3.291	140.8	2.3	1.6	2.8	6.3	6.1	6.4	2.0	ARGGGTFWNS<br>VYYFDY	QVQLVQSGAEVKKPGSSVKVSCKASGGTFSSY<br>AISWVRQAPGQGLEWMGGIIPIFHTANYAQKF<br>QGRVTITADESTSTAYMELSSLRSEDTAVYYC<br>ARGGGTFWNSVYYFDYWGQGTLVTVSS
D339_2W1_701853	3.498	0.013	-	-	-	-	-	-	-	-	ARGGGTLLEFG<br>YYFDY	QVQLVQSGAEVKKPGVSVKVSCKASGGTFSS<br>YAISWVQQAPGQGLEWMGGIIPIFHTANYAQK<br>FQGRVTITADESTSTAYMELSSLRSEDTAVYY<br>CARGGGTLLEFGYYFDYWGQGTLVTVSS
D339_2W1_134156	3.754	4.452	18.564	5.7	3.6	3.4	9.7	9.3	5.1	3.6	ARGGGTFWNS<br>VYYFDY	RWQLVQSGAEVKKPGSSVKVSCKASGGTFSS<br>YAISWVRQAPGQGLEWMGGIIPIFHTANYAQK<br>FQGRVTITADESTSTAYMELSSLRSEDTAVYY<br>CARGGGTFWNSVYYFDYWGQGTLVTVSS
D339_2W1_1218854	2.659	2.659	19.8	9.4	3.1	4.1	10.2	6.8	6.1	7.9	ARGGGTFWNS<br>VYYFDY	GVHGVQSGAEVKKPGSSVKVSCKASGGTFSS<br>YAISWVRQAPRQGLEWMGGIIPIFHTANYAQK<br>FQGRVTITADESTSTAYMELSSLRSEDTAVYY<br>CARGGGTFWNSVYYFDYWGQGTLVTVSS
D339_2W1_1286967	3.449	3.815	63	11.2	3.7	7.9	16.3	16.3	8.4	8.0	ARGGETFWNS<br>VYYFDY	QVQLVQSGAEVKKPGSSVKVSCKASGGTFSSY<br>AISWVRQAPGQGLEWMGGIIPIFHTANYAQKF<br>QGRVTITADESTSTAYMELSSLRSEDTAVYYC<br>ARGGETFWNSVYYFDYWGQGTLVTVSS
D339_2W1_416870	1.799	0.003	-	-	-	-	-	-	-	-	ARGGGRSGIRF<br>SYFDY	STVGWQSGAEVENAGASVKVSCKASGGTFSS<br>YAISWVRQAPAQGLEWMGGIIPIFRTANYAQK

												FQGRVTITADESTSTAYMELSSLRSEDTAVYY CARGGGRSGIRFSYFDYWGQGTLVTVSS
D339_3W1_663148	2.970	3.512	3.6	0.5	0.6	0.7	6.6	2.0	0.9	8.2	ARGGGTFWNS VYYFDY	RYHGVQSGAEVKKPGSSVKVSCKASGGTFSS YAISWVRQAPRQGLEWMGGIIPIFHTANYAQK FQGRVTITADESTSTAYMELSSLRSEDTAVYY CARGGGTFWNSVYYFDYWGQGTLVTVSS
D355_2W1_101394	0.028	0.011	-	-	-	-	-	-	-	-	ARGPLYGYWY V	VQLQAKWGAGLLKPSETLSLTCAVYGGSFSG YYWSWIPDPPGRGLEWIGEVNQLTGSTNNNPS LKSRVTISVDTSKNQFSLTLSSVTAADTAVYY CARGPLYGYWYVWGQGPTVTVSS
D355_2W1_1226894	2.899	3.456	2.5	>1000	>1000	>1000	>1000	>1000	>1000	>1000	ARGPLYGMDV	RVQLQQWGAGLLKPSETLSLTCAVYGGSFSG YYWSWIRQPPGKGLEWIGEVNHTGSTNYNPS LKSRVTISVDTSKNQFSLTLSSVAAADTAVYY CARGPLYGMDVWGQGTTVTVSS
D355_2W1_1325889	1.803	0.017	-	-	-	-	-	-	-	-	ARGPLYGTGSS	QVQLQQWGAGLLKPSETLSLTCAVYGGSFSG YYWSWIRQPPGKGLEWIGEVNHTGSTNYNPS LKSRVTISVDTSKNQFSLTLGSVTAADTAVYY CARGPLYGTGSSGGQGTPVTVSS
D355_2W1_459560	3.425	3.789	22.26	>1000	>1000	>1000	>1000	>1000	>1000	>1000	ARGPVYGMDV	QVQLQQWGAGLLKPSETLSLTCAVYGGSFSG YYWSWIRQPPGKGLEWIGEVNHTGSTNYNPS LKSRVTISVDTSKNQFSLTLSSVTAADTAVYY CARGPVYGMDVWGQGTTVTVSS

[a] For heavy chain variants, the nomenclature is defined as [Parental antibody]_[Time point]_[Sequence index].

[b] Only HEV p239(1)-specific antibodies were analyzed for protein yield and reactivity to different genotypes HEV capsid proteins.

Supplementary Table 8. Primer sequences for single-cell PCR.

Primer Name	Primer sequence (5'-3')
5' L-VH 1	ACAGGTGCCCACTCCCAGGTGCAG
5' L-VH 3	AAGGTGTCCAGTGTGARGTGCAG
5' L-VH 4/6	CCCAGATGGGTCCTGTCCCAGGTGCAG
5' L-VH 5	CAAGGAGTCTGTTCCGAGGTGCAG
3' Cγ CH1	GGAAGGTGTGCACGCCGCTGGTC
5' AgeI VH1	CTGCAACCGGTGTACATTCCCAGGTGCAGCTGGTGCAG
5' AgeI VH1/5	CTGCAACCGGTGTACATTCCGAGGTGCAGCTGGTGCAG
5' AgeI VH3	CTGCAACCGGTGTACATTCTGAGGTGCAGCTGGTGGAG
5' AgeI VH3-23	CTGCAACCGGTGTACATTCTGAGGTGCAGCTGTTGGAG
5' AgeI VH4	CTGCAACCGGTGTACATTCCCAGGTGCAGCTGCAGGAG
5' AgeI VH 4-34	CTGCAACCGGTGTACATTCCCAGGTGCAGCTACAGCAGTG
5' AgeI VH 1-18	CTGCAACCGGTGTACATTCCCAGGTTCAGCTGGTGCAG
5' AgeI VH 1-24	CTGCAACCGGTGTACATTCCCAGGTCCAGCTGGTACAG
5' AgeI VH3-33	CTGCAACCGGTGTACATTCTCAGGTGCAGCTGGTGGAG
5' AgeI VH 3-9	CTGCAACCGGTGTACATTCTGAAGTGCAGCTGGTGGAG
5' AgeI VH4-39	CTGCAACCGGTGTACATTCCCAGCTGCAGCTGCAGGAG
5' AgeI VH 6-1	CTGCAACCGGTGTACATTCCCAGGTACAGCTGCAGCAG
3' IgG (internal)	GTTCGGGGAAGTAGTCCTTGAC
5' L Vκ 1/2	ATGAGGSTCCCYGCTCAGCTGCTGG
5' L Vκ 3	CTCTTCCTCCTGCTACTCTGGCTCCCAG
5' L Vκ 4	ATTTCTCTGTTGCTCTGGATCTCTG
3' Cκ 543	GTTTCTCGTAGTCTGCTTTGCTCA
5' Pan Vκ	ATGACCCAGWCTCCABYCWCCCTG
3' Cκ 494	GTGCTGTCCTTGCTGTCCTGCT
5' AgeI Vκ 1-5	CTGCAACCGGTGTACATTCTGACATCCAGATGACCCAGTC
5' AgeI Vκ 1-9	TTGTGCTGCAACCGGTGTACATTCAGACATCCAGTTGACCCAGTCT
5' AgeI Vκ 1D-43	CTGCAACCGGTGTACATTGTGCCATCCGGATGACCCAGTC
5' AgeI Vκ 2-24	CTGCAACCGGTGTACATGGGGATATTGTGATGACCCAGAC
5' AgeI Vκ 2-28	CTGCAACCGGTGTACATGGGGATATTGTGATGACTCAGTC
5' AgeI Vκ 2-30	CTGCAACCGGTGTACATGGGGATGTTGTGATGACTCAGTC
5' Age Vκ 3-11	TTGTGCTGCAACCGGTGTACATTCAGAAATTGTGTTGACACAGTC
5' Age Vκ 3-15	CTGCAACCGGTGTACATTCAGAAATAGTGATGACGCAGTC
5' Age Vκ 3-20	TTGTGCTGCAACCGGTGTACATTCAGAAATTGTGTTGACGCAGTCT
5' Age Vκ 4-1	CTGCAACCGGTGTACATTCGGACATCGTGATGACCCAGTC
5' L Vλ 1	GGTCCTGGGCCCAGTCTGTGCTG
5' L Vλ 2	GGTCCTGGGCCCAGTCTGCCCTG
5' L Vλ 3	GCTCTGTGACCTCCTATGAGCTG
5' L Vλ 4/5	GGTCTCTCTCSCAGCYTGTGCTG
5' L Vλ 6	GTTCTTGGGCCAATTTTATGCTG
5' L Vλ 7	GGTCCAATTCYCAGGCTGTGGTG
5' L Vλ 8	GAGTGGATTCTCAGACTGTGGTG
3' Cλ	CACCAGTGTGGCCTTGTTGGCTTG
5' AgeI Vλ 1	CTGCTACCGGTTCCTGGGCCCAGTCTGTGCTGACKCAG
5' AgeI Vλ 2	CTGCTACCGGTTCCTGGGCCCAGTCTGCCCTGACTCAG
5' AgeI Vλ 3	CTGCTACCGGTTCTGTGACCTCCTATGAGCTGACWCAG
5' AgeI Vλ 4/5	CTGCTACCGGTTCTCTCTCSCAGCYTGTGCTGACTCA
5' AgeI Vλ 6	CTGCTACCGGTTCTTGGGCCAATTTTATGCTGACTCAG
5' AgeI Vλ 7/8	CTGCTACCGGTTCCAATTCYCAGRCTGTGGTGACYCAG
3' XhoI Cλ	CTCCTCACTCGAGGGYGGGAACAGAGTG
3' Sall JH 1/2/4/5	TGCGAAGTCGACGCTGAGGAGACGGTGACCAG
3' Sall JH 3	TGCGAAGTCGACGCTGAAGAGACGGTGACCATTG
3' Sall JH 6	TGCGAAGTCGACGCTGAGGAGACGGTGACCGTG
3' BsiWI Jκ 1/4	GCCACCGTACGTTTGATYTCCACCTTGGTC
3' BsiWI Jκ 2	GCCACCGTACGTTTGATCTCCAGCTTGGTC

3' BsiWI Jκ 3	GCCACCGTACGTTTGATATCCACTTTGGTC
3' BsiWI Jκ 5	GCCACCGTACGTTTAATCTCCAGTCGTGTC

Supplementary Table 9. 5'-RACE PCR primers used to prepare samples for Ion S5/PGM sequencing.

Chains	Primer Name	Primer sequence (5'-3')
Heavy chain	trP1/P1-5'-RACE	CCTCTCTATGGGCAGTCGGTGAT 5'-RACE adaptor
	VRC3'-A-CγCH1	CCATCTCATCCCTGCGTGTCTCCGACTCAG GGGGAAGACCGATGGGCCCTTGGTGG
	VRC3'-A-CμCH1	CCATCTCATCCCTGCGTGTCTCCGACTCAG GGGAATTCTCACAGGAGACGA
Lamada chain	trP1/P1-5'-RACE	CCTCTCTATGGGCAGTCGGTGAT 5'-RACE adaptor
	VRC3'-A-Cλ	CCATCTCATCCCTGCGTGTCTCCGACTCAG CACCAGTGTGGCCTTGTTGGCTTG
Kappa chain	trP1/P1-5'-RACE	CCTCTCTATGGGCAGTCGGTGAT 5'-RACE adaptor
	VRC3'-A-Cκ	CCATCTCATCCCTGCGTGTCTCCGACTCAG CAGCAGGCACACAACAGAGGCAGTTCC

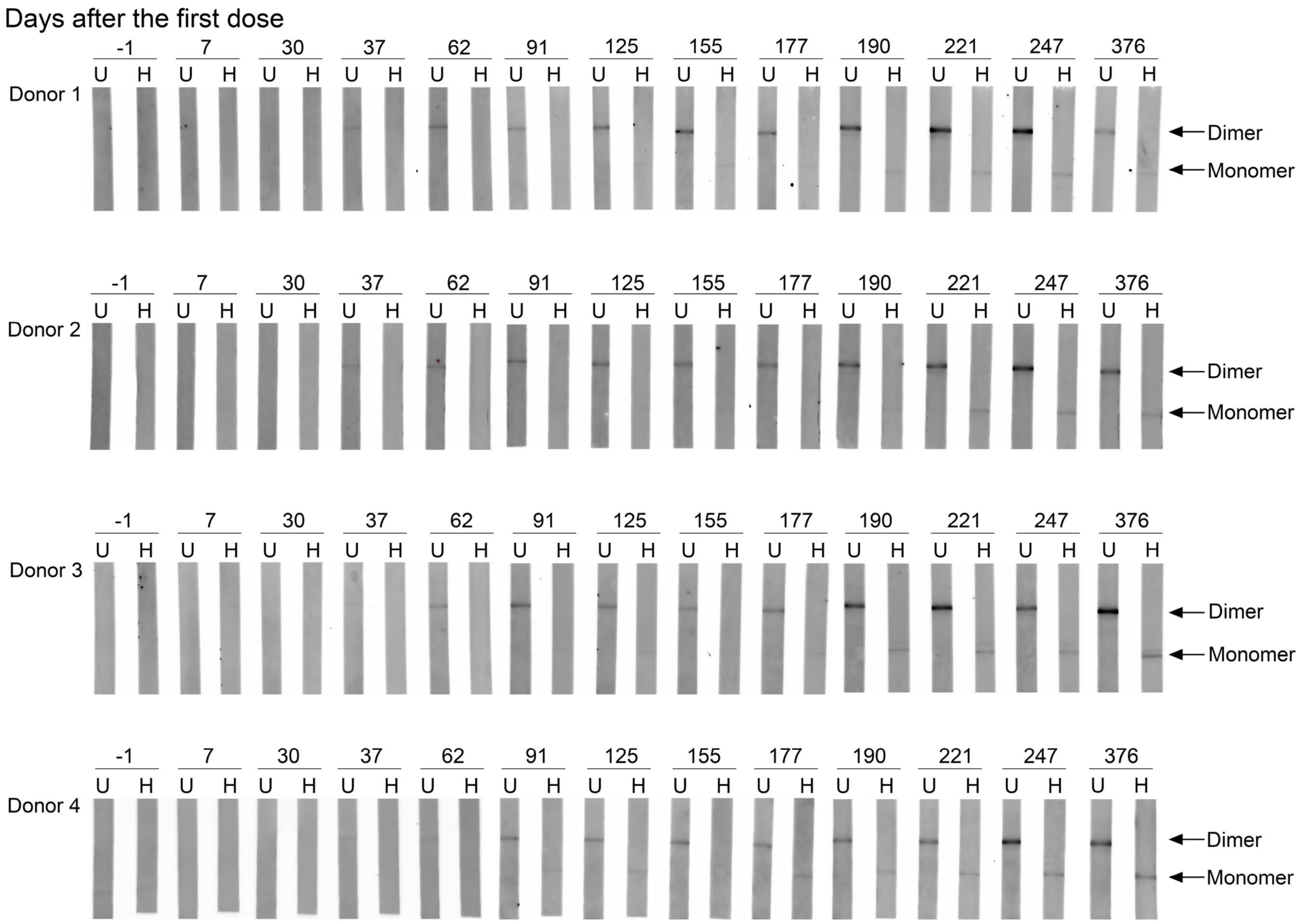

Supplementary Figure 1. Analysis of the sera collected at different time points during the vaccination by western blot. Samples of p239(1) were subjected to western blot with the sera to investigate their reactivity. The lanes marked with H indicate heated samples. The lanes marked with U indicate unheated samples. The time of the sera collection (days after the first dose) are indicated above the horizontal line. The first, second, and third doses are indicated above the time axis at days 0, 31, and 183, respectively. This experiment was performed for one time to obtain all the necessary data. Source data are provided as a Source Data file.

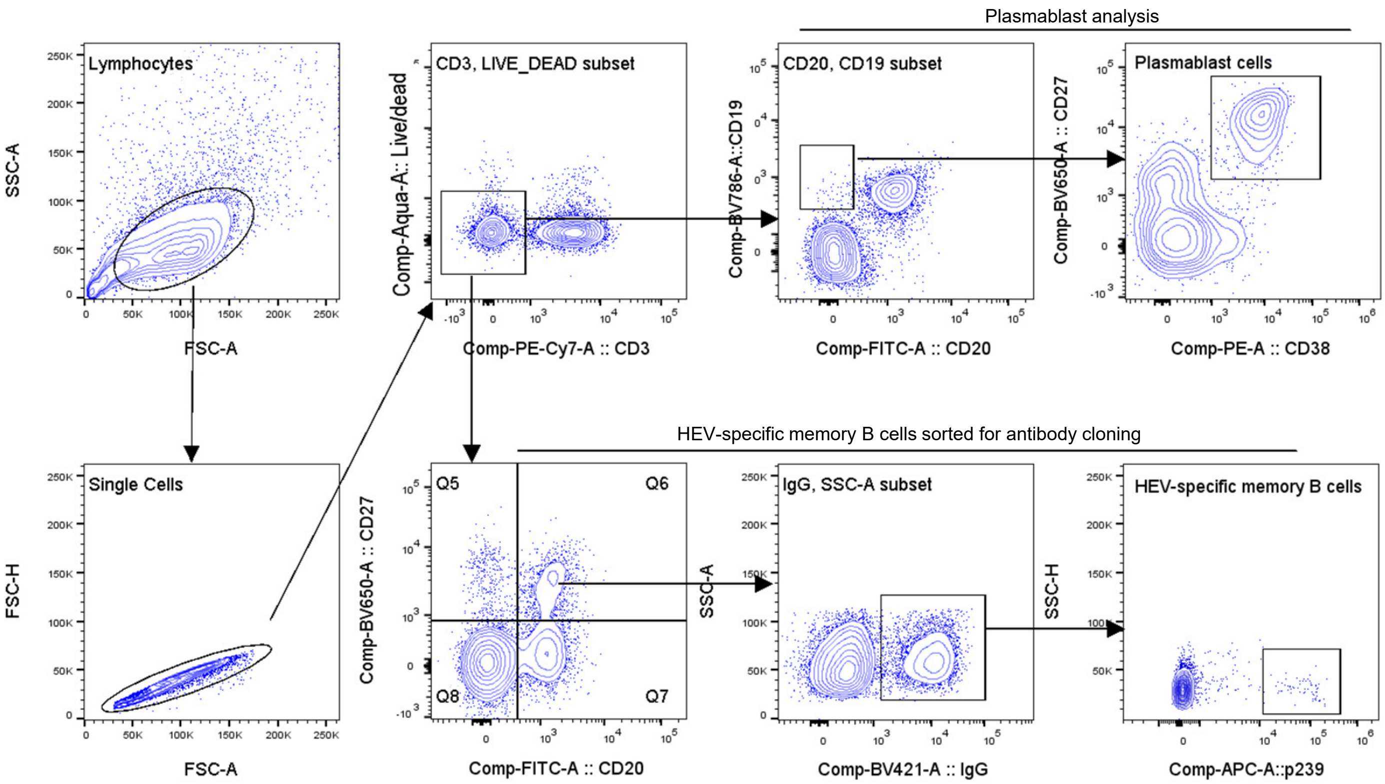

Supplementary Figure 2. Representative gating strategy for plasmablasts and IgG+ HEV p239(1)-specific memory B cells. Peripheral blood mononuclear cells (PBMCs) were stained as described in the Materials and Methods. CD3-/CD20-/CD19+/CD27+/CD38+ cells were defined as plasmablasts. Based on the gating strategy, dynamics of plasmablasts was analyzed and presented in Supplementary Figure 3. CD3-/CD20+/CD27+/IgG+ cells were defined as IgG+ memory B cells. IgG+ memory B cells that showed reactivity with HEV p239(1) were single-cell sorted for antibody cloning and presented in Supplementary Figure 4.

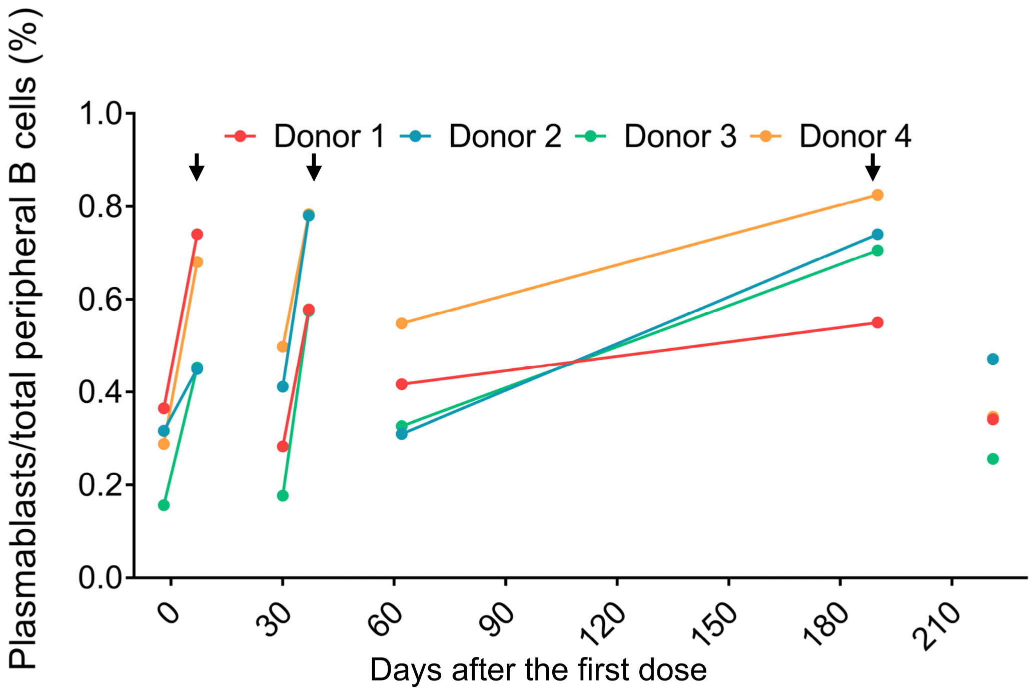

Supplementary Figure 3. The ratio of plasmablasts versus total peripheral B cells in peripheral blood mononuclear cells (PBMCs) collected at seven time points during the vaccination. CD3-/CD20-/CD19+/CD27+/CD38+ cells were defined as plasmablasts. Donors 1, 2, 3, and 4 are indicated by red, cyan, green, and orange, respectively. Source data are provided as a Source Data file.

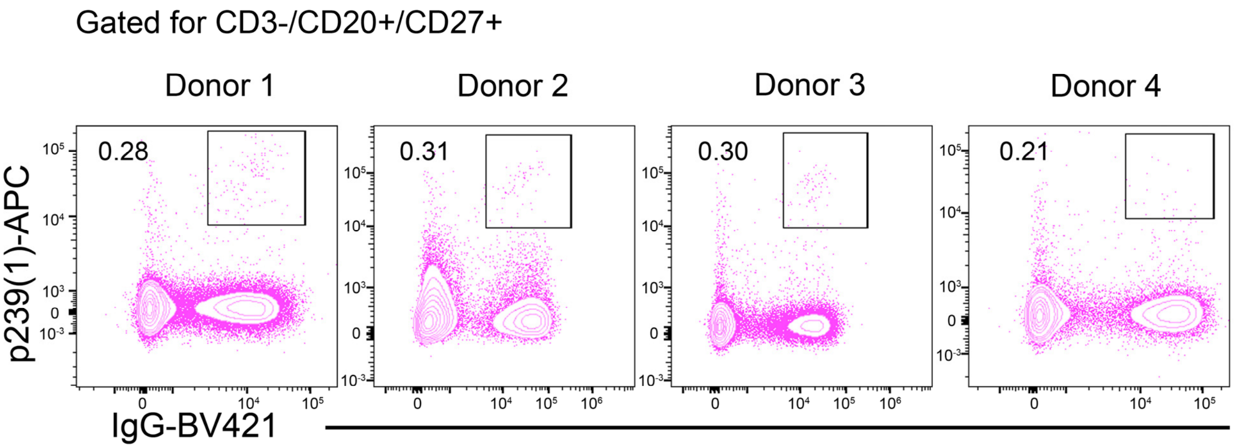

Supplementary Figure 4. Percentage of memory B cells that were IgG+ HEV p239(1)-specific in PBMCs collected one month after the third dose (3M1) in four donors. CD3-/CD20+/CD27+ cells were defined as memory B cells. The percentage of IgG+ HEV p239(1)-specific memory B cells were indicated. Source data are provided as a Source Data file.

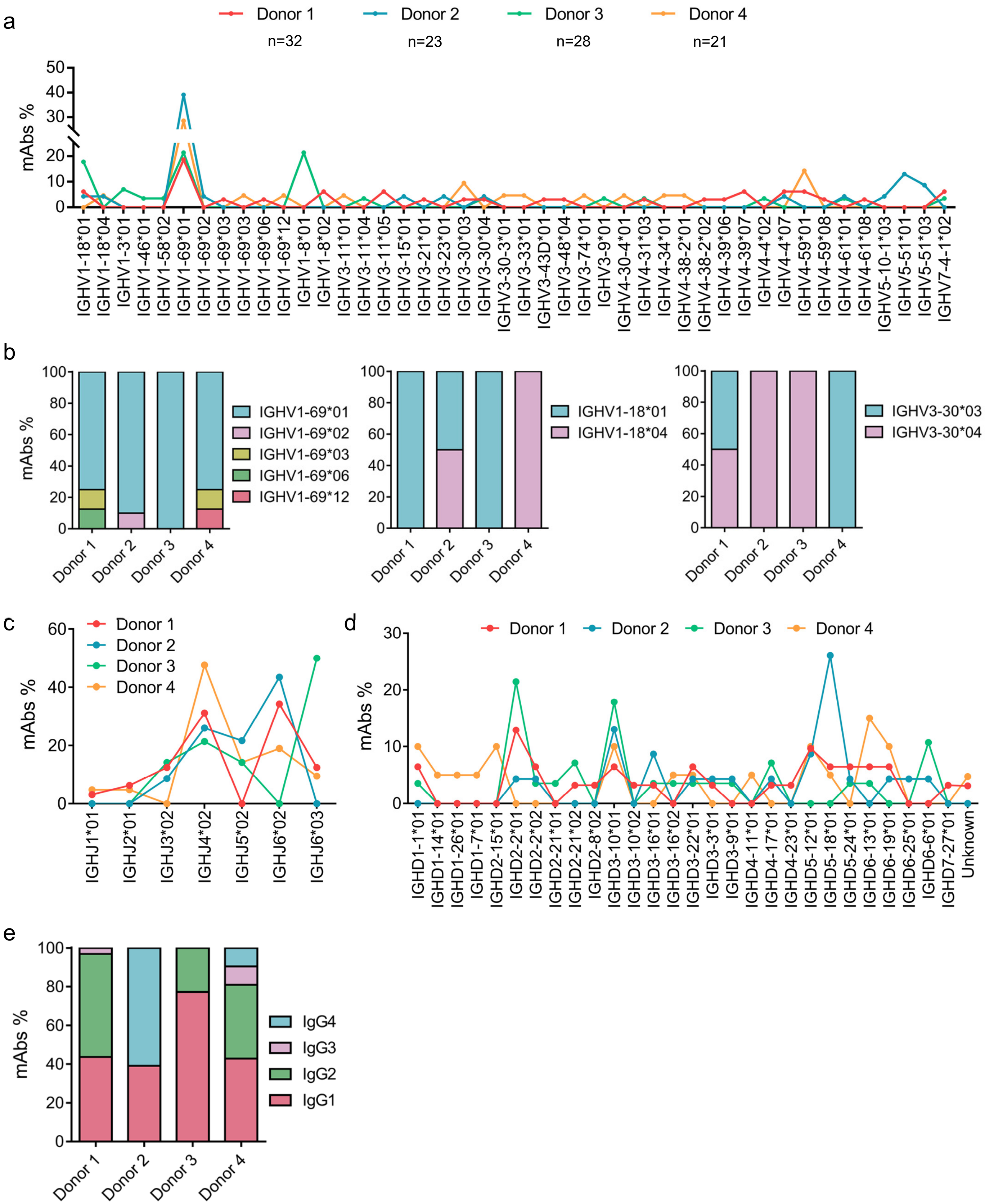

Supplementary Figure 5. Distribution of heavy-chain germline genes and alleles. (**a**) Distribution of heavy-chain variable (VH) germline genes and alleles. (**b**) Distribution of alleles variants in IGHV1-69, IGHV1-18, IGHV3-30. (**c**) Distribution of heavy-chain joining (JH) germline genes and alleles. (**d**) Distribution of heavy-chain diversity (DH) germline genes and alleles. Donors 1, 2, 3, and 4 are indicated by red, cyan, green, and orange in (**a**), (**c**) and (**d**), respectively. (**e**) IgG subtype analysis. Six heavy chains from donor 3 were excluded in this analysis because their subtypes could not be reliably determined based on their sequences. Source data are provided as a Source Data file.

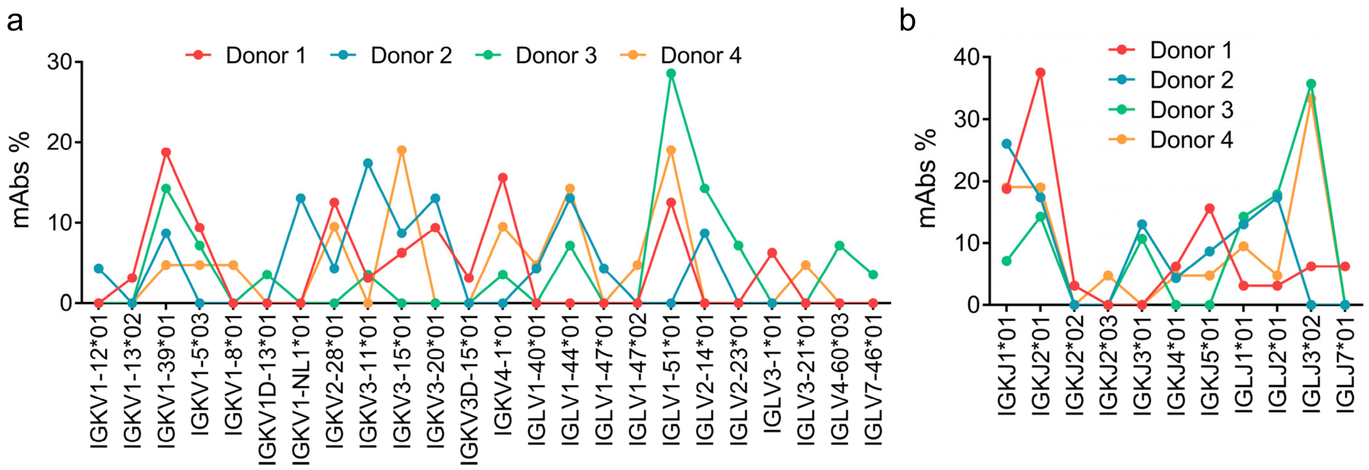

Supplementary Figure 6. Distribution of light-chain germline genes and alleles. (**a**) Distribution of light-chain variable (VL) germline genes and alleles. (**b**) Distribution of light-chain joining (JL) germline genes and alleles. Donors 1, 2, 3, and 4 are indicated by red, cyan, green, and orange, respectively. Source data are provided as a Source Data file.

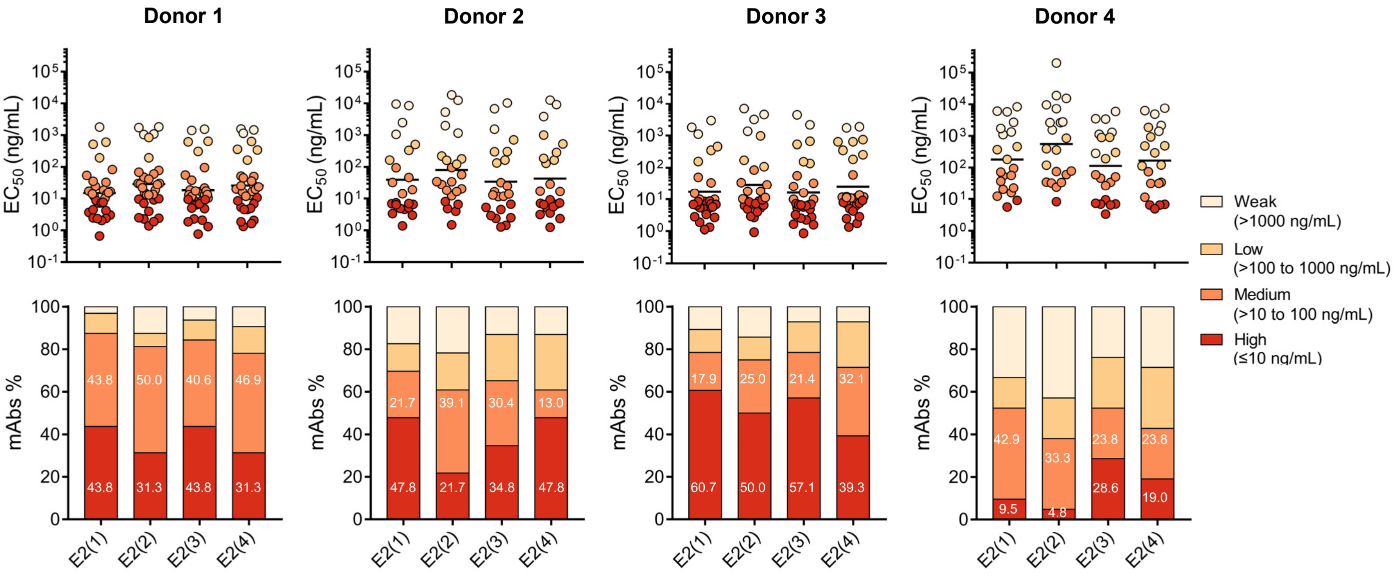

Supplementary Figure 7. Binding activity of HEV p239(1)-specific mAbs with the E2 proteins of four genotypes. Upper panel: the binding activity (EC_{50}) of HEV p239(1)-specific mAbs measured for the E2 proteins of genotypes 1, 2, 3, and 4 in four ranges (weak, low, medium, and high); Lower panel: the percentage of mAbs within the indicated EC_{50} range shown for four genotypes. Of note, all mAbs can recognize the E2 protein across four genotypes, despite their different binding activities. Each cycle represented a mAb. There were 32 mAbs per group in donor 1, 23 mAbs per group in donor 2, 28 mAbs per group in donor 3, and 21 mAbs per group in donor 4. Source data are provided as a Source Data file.

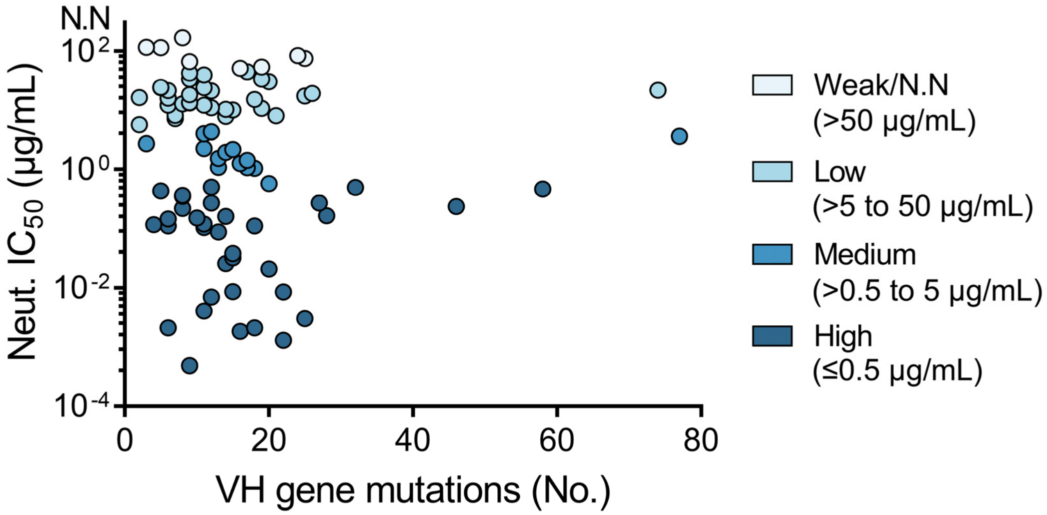

Supplementary Figure 8. Relationship between somatic hypermutation (SHM) and neutralizing activity. The degree of SHM does not correlate with neutralizing activity. Neutralization IC_{50} is plotted against the number of heavy-chain variable (VH) nucleotide substitutions for each mAb, which is colored according to the neutralizing activity (IC_{50}) of four ranges. N.N., non-neutralizing. Of note, since non-neutralizing mAbs were excluded from the statistical analysis, they are not shown on this plot. Eighty-eight mAbs were shown in this figure. Spearman rank correlation analysis were used to correlation between SHM and HEV neutralization, (p=0.191, r=-0.141). Source data are provided as a Source Data file.

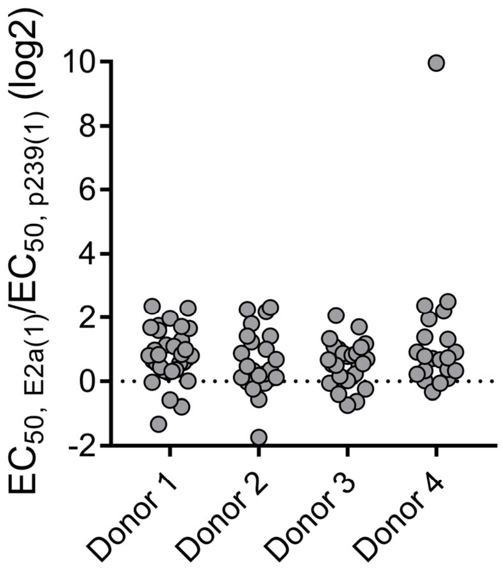

Supplementary Figure 9. Comparison of binding activity of HEV p239(1)-specific mAbs with the E2a(1) and p239(1) proteins. To overcome the low stability of recombinant E2s, we tested stable p239(1) (aa 368-606) and E2a(1) (aa 459-660), which both contain E2s. The data were processed and transformed using the formula log2 (EC_{50} E2a(1)/EC_{50} p239(1)). The mAbs showed similar binding affinity for both HEV antigens would be considered capable of recognizing the E2s domain (aa 459-606). Overall, HEV p239(1)-specific mAbs demonstrated comparable binding affinity for E2a(1) and p239(1). There were 32 mAbs in donor 1, 23 mAbs in donor 2, 28 mAbs in donor 3, and 21 mAbs in donor 4. Source data are provided as a Source Data file.

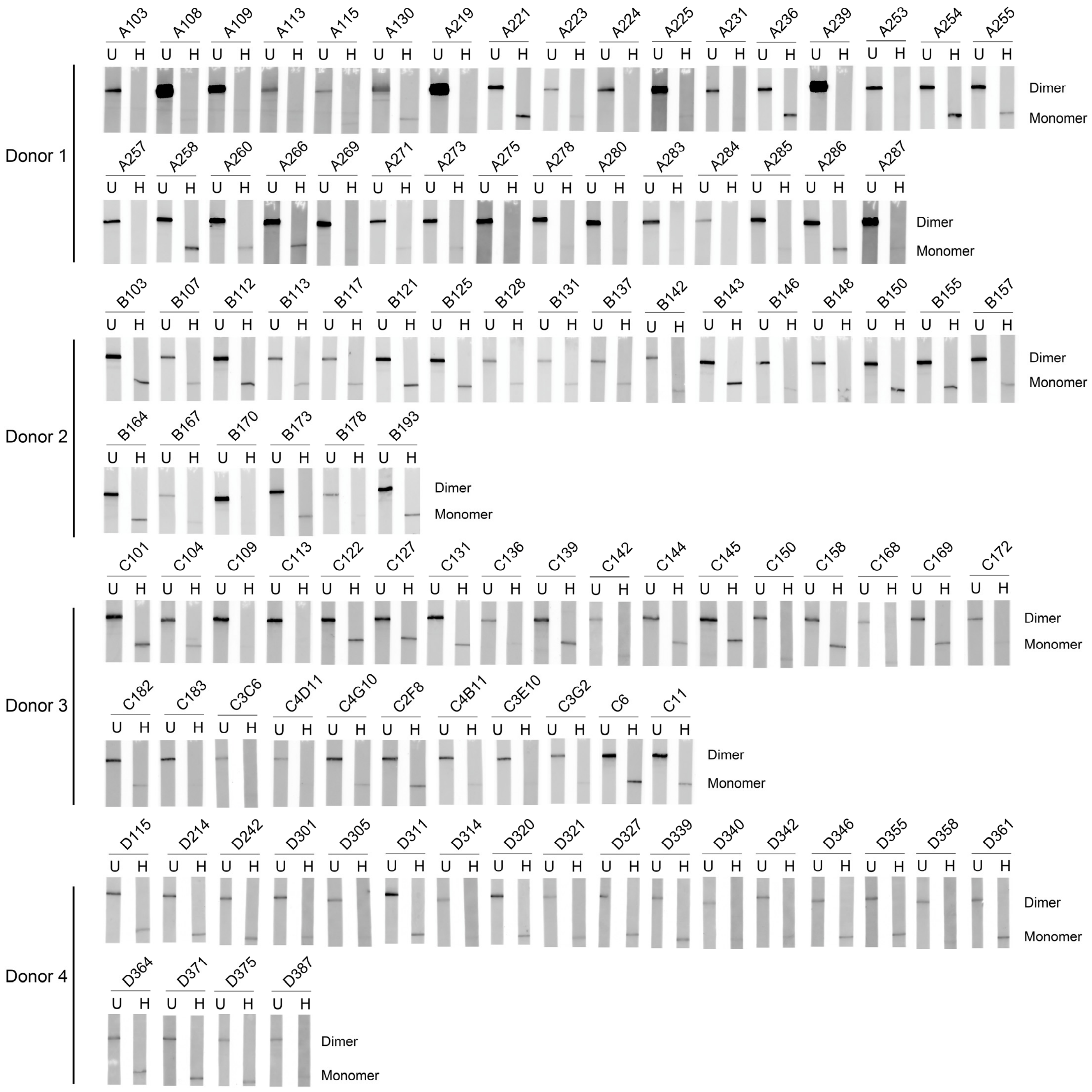

Supplementary Figure 10. Analysis of HEV p239(1)-specific mAbs by western blot. Samples of p239(1) with (H) or without boiling (U) were probed with these mAbs. For all mAbs, the binding affinity for the dimeric form of p239(1) was greater than that for the monomeric form of p239(1). This experiment was performed for one time to obtain all the necessary data. Source data are provided as a Source Data file.

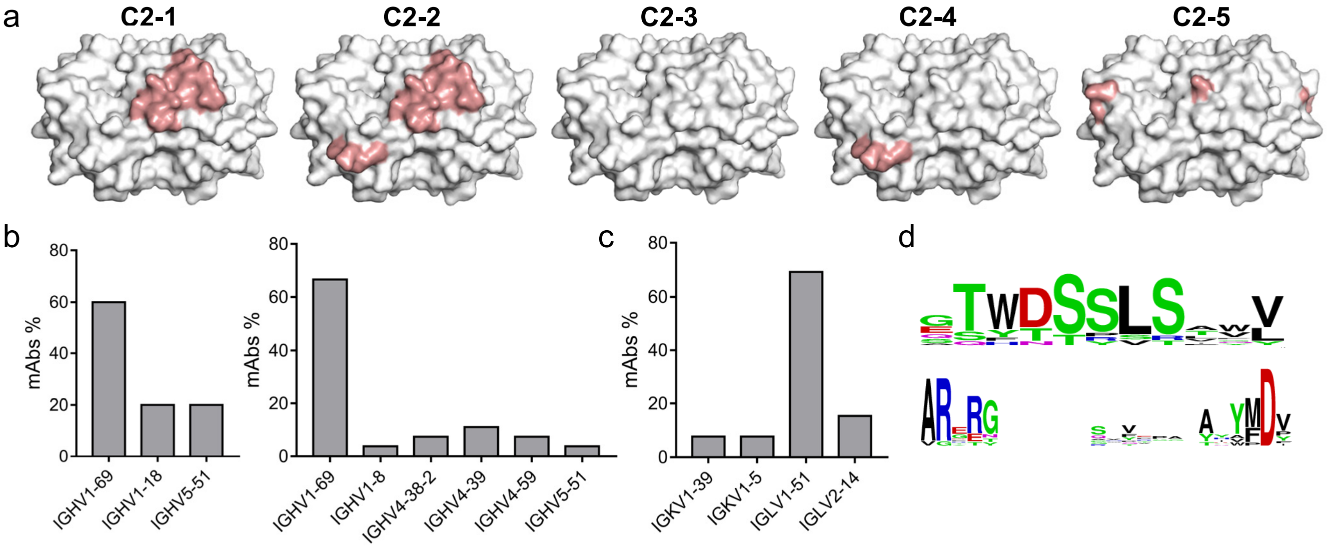

Supplementary Figure 11. Germline and sequence features of HEV p239(1)-specific mAbs targeting antigenic sites C2 and C6. (**a**) Location of five subtypes of antigen site C2 on the protruding domain of the HEV capsid. (**b**) Usage of heavy-chain variable (VH) germline genes for antigen site C2-2 and C2-3 directed mAbs. (**c**) Usage of light chain variable (VL) germline genes for antigen site C6-directed mAbs. (**d**) WebLogos of CDRL3 (Top) and CDRH3 (Bottom) sequences for antigen site C6-directed mAbs. Source data are provided as a Source Data file.

a The frequencies of eight major VH germline genes

Donor 1

	Pre (%)	1W1 (%)	1M1 (%)	2W1 (%)	2M1 (%)	3W1 (%)	3M1 (%)
IGHV1-18	1.39	4.02	4.85	4.31	5.00	1.07	2.73
IGHV1-2	5.35	5.98	4.37	4.19	3.56	2.10	4.98
IGHV1-69	1.66	2.61	4.38	3.74	2.52	2.26	4.20
IGHV3-23	10.72	8.57	5.57	8.75	11.39	13.49	8.31
IGHV3-7	18.62	10.89	3.91	7.22	9.11	10.95	8.10
IGHV4-34	4.78	7.46	12.91	6.92	4.06	5.43	2.80
IGHV4-39	16.64	10.93	16.32	10.74	12.72	27.43	14.69
IGHV4-59	2.77	3.71	4.37	4.84	5.54	3.26	7.99
Sum	61.93	54.17	56.67	50.72	53.89	65.99	53.78

Donor 2

	Pre (%)	1W1 (%)	1M1 (%)	2W1 (%)	2M1 (%)	3W1 (%)	3M1 (%)
IGHV1-18	3.91	2.53	3.54	7.96	3.79	3.37	2.04
IGHV1-2	3.16	9.66	3.83	1.02	3.08	2.19	3.43
IGHV1-69	4.35	4.19	7.79	12.48	6.18	15.26	8.00
IGHV3-23	6.60	11.05	4.75	6.77	7.36	10.26	9.71
IGHV3-7	7.99	13.82	6.67	1.17	6.25	4.35	11.78
IGHV4-34	3.53	3.90	5.08	19.66	3.60	9.37	7.94
IGHV4-39	10.52	11.55	15.71	5.11	10.95	12.29	13.01
IGHV4-59	2.79	2.76	4.02	2.69	6.15	5.81	2.66
Sum	42.83	59.45	51.37	56.86	47.35	62.90	58.58

Donor 3

	Pre (%)	1W1 (%)	1M1 (%)	2W1 (%)	2M1 (%)	3W1 (%)	3M1 (%)
IGHV1-18	4.33	2.57	4.48	4.10	4.28	8.02	4.26
IGHV1-2	3.08	2.47	3.56	4.59	2.65	2.82	2.62
IGHV1-69	4.02	11.60	3.55	1.58	4.27	4.73	3.70
IGHV3-23	13.13	7.99	8.80	9.05	7.96	6.28	10.55
IGHV3-7	9.24	5.89	11.54	12.81	10.95	7.39	12.56
IGHV4-34	2.50	1.80	2.26	2.33	2.81	3.64	2.64
IGHV4-39	9.43	7.93	7.85	9.53	7.30	7.52	7.07
IGHV4-59	3.16	5.32	3.91	3.69	3.80	5.40	2.53
Sum	48.89	45.57	45.96	47.66	44.03	45.80	45.91

Donor 4

	Pre (%)	1W1 (%)	1M1 (%)	2W1 (%)	2M1 (%)	3W1 (%)	3M1 (%)
IGHV1-18	1.37	10.15	2.23	6.70	4.79	2.58	2.29
IGHV1-2	7.09	3.83	5.82	5.95	3.96	6.28	7.78
IGHV1-69	2.46	4.71	3.23	5.12	2.03	11.79	2.95
IGHV3-23	8.37	4.54	8.07	6.90	11.96	3.71	7.77
IGHV3-7	15.52	8.31	15.41	7.67	10.06	1.83	16.73
IGHV4-34	5.01	12.25	4.79	3.22	6.17	2.62	3.03
IGHV4-39	15.28	12.83	9.19	13.55	8.54	17.79	13.08
IGHV4-59	3.96	2.47	4.43	8.10	4.64	4.67	3.85
Sum	59.06	59.08	53.18	57.20	52.14	51.27	57.47

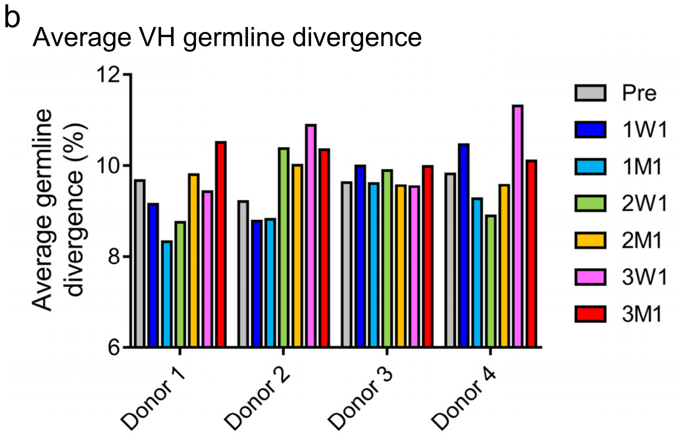

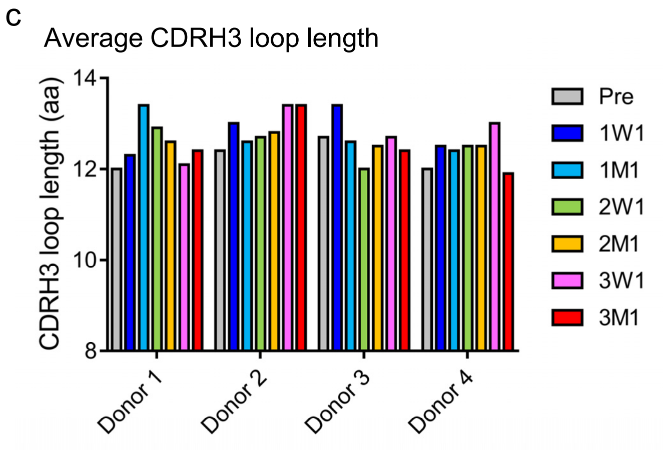

Supplementary Figure 12. Unbiased B cell repertoire profiles of four donors at seven time points during the vaccination. (**a**) The frequencies of eight major heavy-chain variable (VH) germline genes in the B cell repertoire, (**b**) average VH germline divergence, or degree of SHM, and (**c**) average CDRH3 loop length. Pre: pre-vaccination; 1W1: one week after the first dose; 1M1: one month after the first dose; 2W1: one week after the second dose; 2M1: one month after the second dose; 3W1: one week after the third dose; 3M1: one month after the third dose. The color-coding scheme is as following: Pre (gray), 1W1 (blue), 1M1 (cyan), 2W1 (green), 2M1 (orange), 3W1 (purple), and 3M1 (red). An oscillating pattern of germline divergence was observed for three donors (1, 2 and 4), suggesting cycles of antibody elicitation and maturation following three vaccine doses. In contrast, donor 3 showed a steady level of SHM, on average 9.5-10.0%, despite the significant shift in germline gene usage during the vaccination. Source data are provided as a Source Data file.

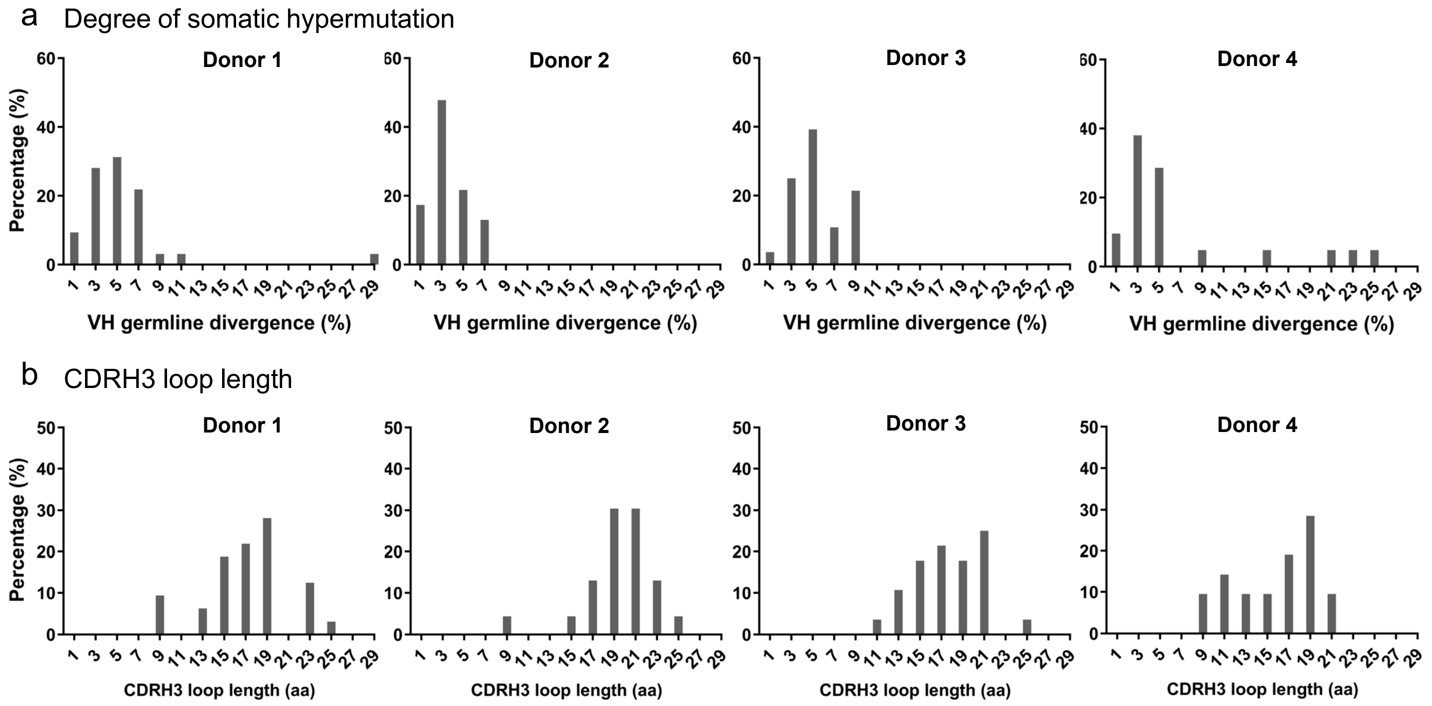

Supplementary Figure 13. Distribution of heavy-chain variable (VH) gene germline divergence and CDRH3 loop length of HEV p239(1)-specific mAbs in four donors. **(a)** VH gene germline divergence, or degree of somatic hypermutation (SHM). **(b)** CDRH3 loop length of HEV p239(1)-specific mAbs. Source data are provided as a Source Data file.

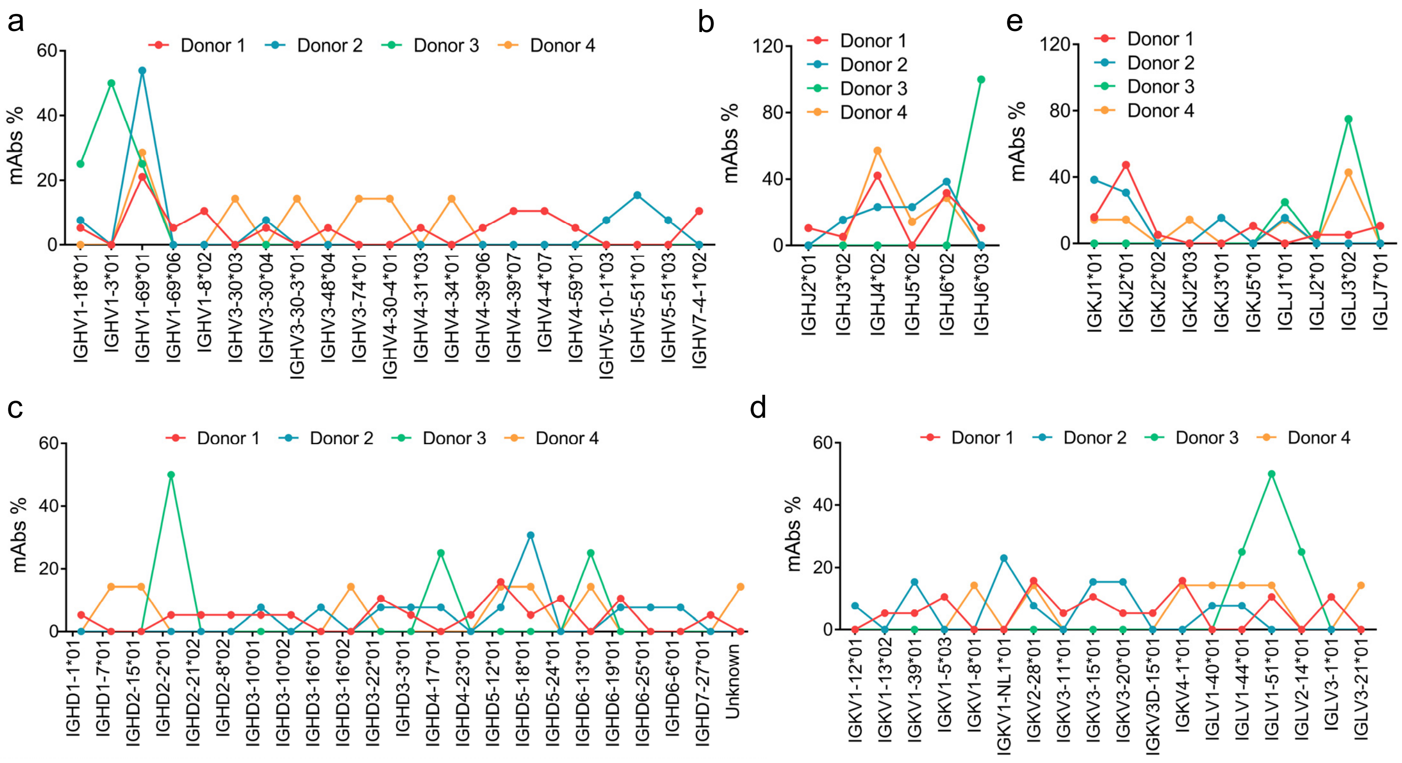

Supplementary Figure 14. Distribution of heavy-chain and light-chain germline genes and alleles. (**a**) Distribution of heavy-chain variable (VH) germline genes and alleles. (**b**) Distribution of heavy-chain joining (JH) germline genes and alleles. (**c**) Distribution of heavy-chain diversity (DH) germline genes and alleles. (**d**) Distribution of light-chain variable (VL) germline genes and alleles. (**e**) Distribution of light-chain joining (JL) germline genes and alleles. Donors 1, 2, 3, and 4 are indicated by red, cyan, green, and orange, respectively. Source data are provided as a Source Data file.

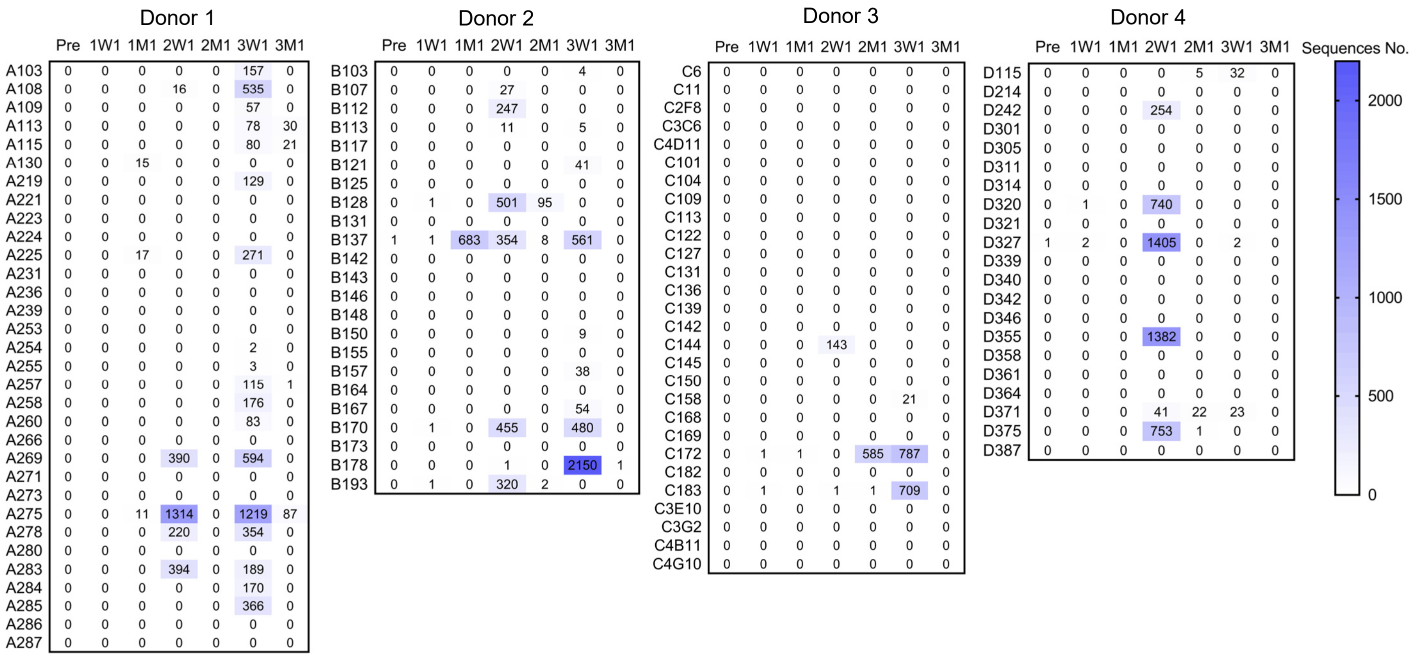

Supplementary Figure 15. Heatmap representation of HEV p239(1)-specific antibody lineage size in the unbiased B cell repertoire for four donors at seven time points during the vaccination. The lineages were defined by a CDRH3 identity of 90% and a CDRH3 length variation of two residues or less. Pre: pre-vaccination; 1W1: one week after the first dose; 1M1: one month after the first dose; 2W1: one week after the second dose; 2M1: one month after the second dose; 3W1: one week after the third dose; 3M1: one month after the third dose. Source data are provided as a Source Data file.

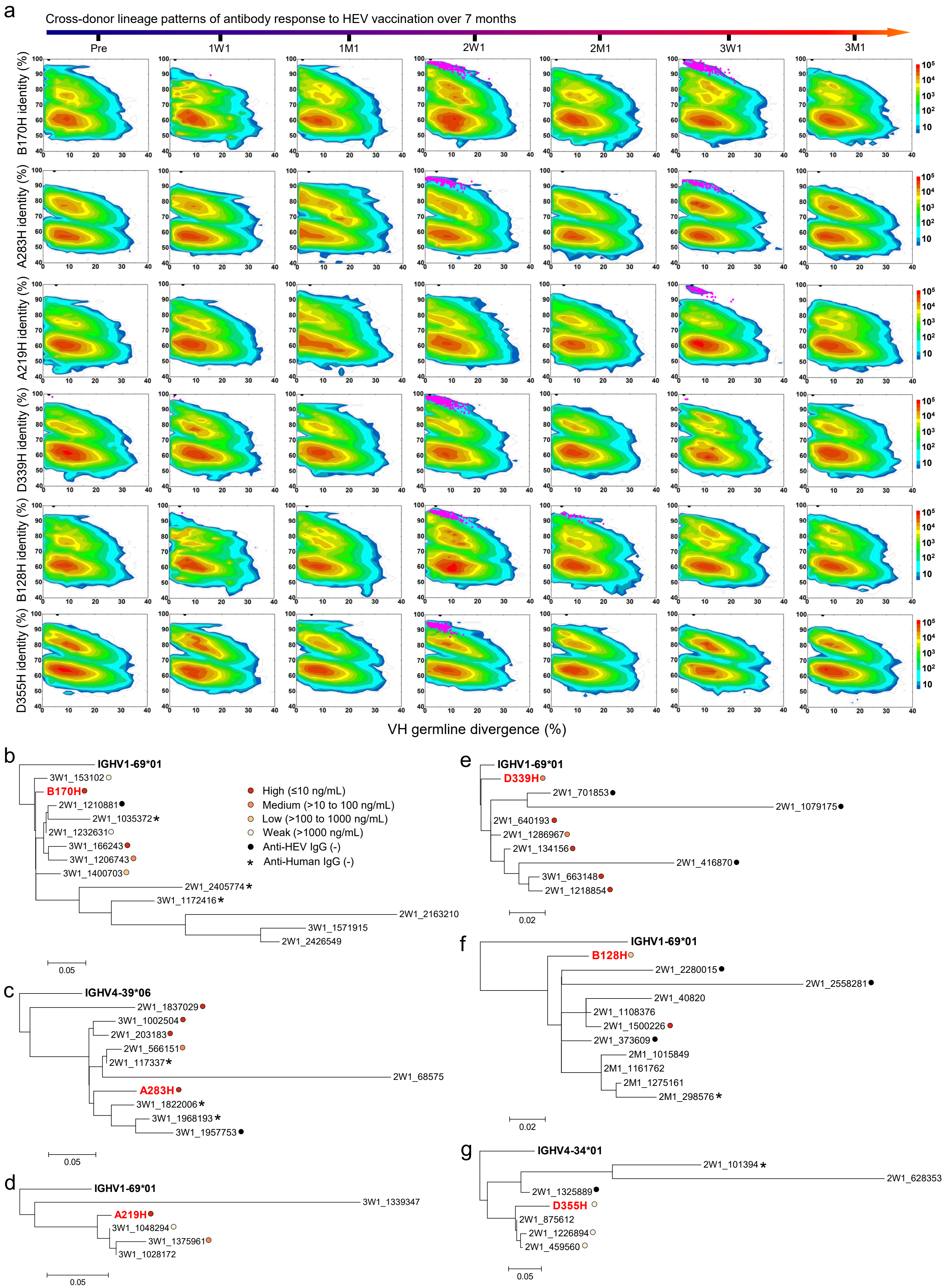

a
Cross-donor lineage patterns of antibody response to HEV vaccination over 7 months
Pre
1W1
1M1
2W1
2M1
3W1
3M1
B170H identity (%)
A283H identity (%)
A219H identity (%)
D339H identity (%)
B128H identity (%)
D355H identity (%)
VH germline divergence (%)
b
IGHV1-69*01
3W1_153102
B170H
2W1_1210881
2W1_1035372
2W1_1232631
3W1_166243
3W1_1206743
3W1_1400703
2W1_2405774
3W1_1172416
2W1_2163210
3W1_1571915
2W1_2426549
0.05
High (≤10 ng/mL)
Medium (>10 to 100 ng/mL)
Low (>100 to 1000 ng/mL)
Weak (>1000 ng/mL)
Anti-HEV IgG (-)
Anti-Human IgG (-)
c
IGHV4-39*06
2W1_1837029
3W1_1002504
2W1_203183
2W1_566151
2W1_117337
2W1_68575
A283H
3W1_1822006
3W1_1968193
3W1_1957753
0.05
d
IGHV1-69*01
3W1_1339347
A219H
3W1_1048294
3W1_1375961
3W1_1028172
0.05
e
IGHV1-69*01
D339H
2W1_701853
2W1_1079175
2W1_640193
2W1_1286967
2W1_134156
2W1_416870
3W1_663148
2W1_1218854
0.02
f
IGHV1-69*01
B128H
2W1_2280015
2W1_2558281
2W1_40820
2W1_1108376
2W1_1500226
2W1_373609
2M1_1015849
2M1_1161762
2M1_1275161
2M1_298576
0.02
g
IGHV4-34*01
2W1_101394
2W1_628353
2W1_1325889
D355H
2W1_875612
2W1_1226894
2W1_459560
0.05

Supplementary Figure 16. Cross-donor patterns of the HEV p239(1)-specific antibody lineage development during the vaccination. (**a**) Identity-divergence plots of the B170, A283, A219, D339, B128, and D355 lineages in the context of unbiased donor B cell repertoires. Heavy chains are plotted as a function of sequence identity to the reference mAbs and sequence divergence (%) from their putative germline genes. Color-coding indicates sequence density on the 2D plot. Somatic variants of B170, A283, A219, D339, B128, and D355 identified by a CDRH3 identity cutoff of 90% are shown as magenta dots on the 2D plots. Pre: pre-vaccination; 1W1: one week after the first dose; 1M1: one month after the first dose; 2W1: one week after the second dose; 2M1: one month after the second dose; 3W1: one week after the third dose; 3M1: one month after the third dose. Dendrograms of selected heavy chain variants from the lineages of B170 (**b**), A283 (**c**), A219 (**d**), D339 (**e**), B128 (**f**), and D355 (**g**) rooted by their putative germline V genes. The NGS-derived somatic variants were paired with light chains of their parental mAbs for functional validation. Reconstituted mAbs are labeled with dots if they can be expressed and bind the HEV vaccine antigen, p239(1). The parental mAb heavy chains is labeled in red on the dendrogram, which was generated using the maximum-likelihood (ML) method. (EC_{50} [ng/mL]: ≤10, red circle; >10 to 100, orange circle; >100 to 1000, aurantia circle; >1000, yellow circle; p239(1)-nonspecific, black).

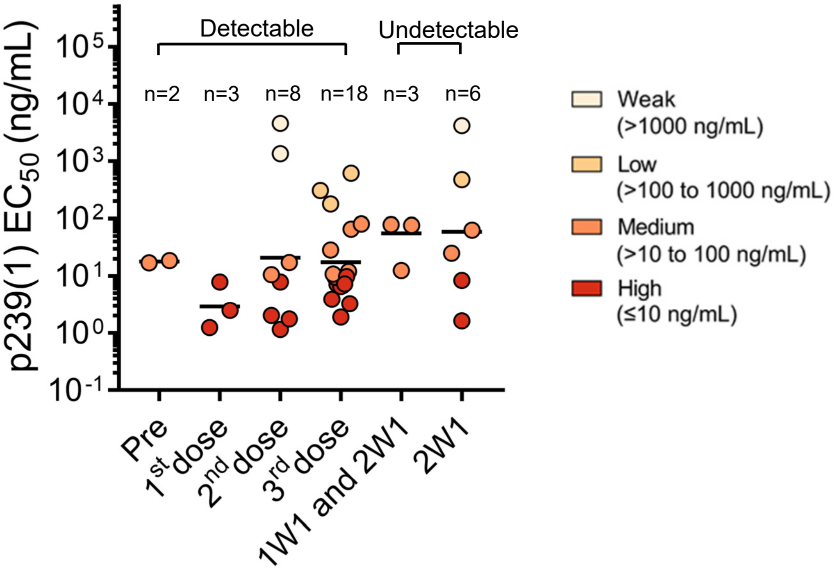

Supplementary Figure 17. Binding activity (EC_{50}) of HEV p239(1)-specific mAbs that exhibit different cross-donor B cell response patterns. The six B cell response patterns are defined as following. Pre: mAbs were first found in the Pre repertoires and showed an evolving B cell response; 1st dose: mAbs were first found in the repertoires after the first dose and showed an evolving B cell response; 2nd dose: mAbs were first found in the repertoires after the second dose and showed an evolving B cell response; 3rd dose: : mAbs were first found in the repertoires after the third dose; 1W1 and 2W1: mAbs could be detected in the 1W1 and 2W1 repertoires; 2W1: mAbs showed a transient B cell response and were only detected in the 2W1 repertoires. Source data are provided as a Source Data file.

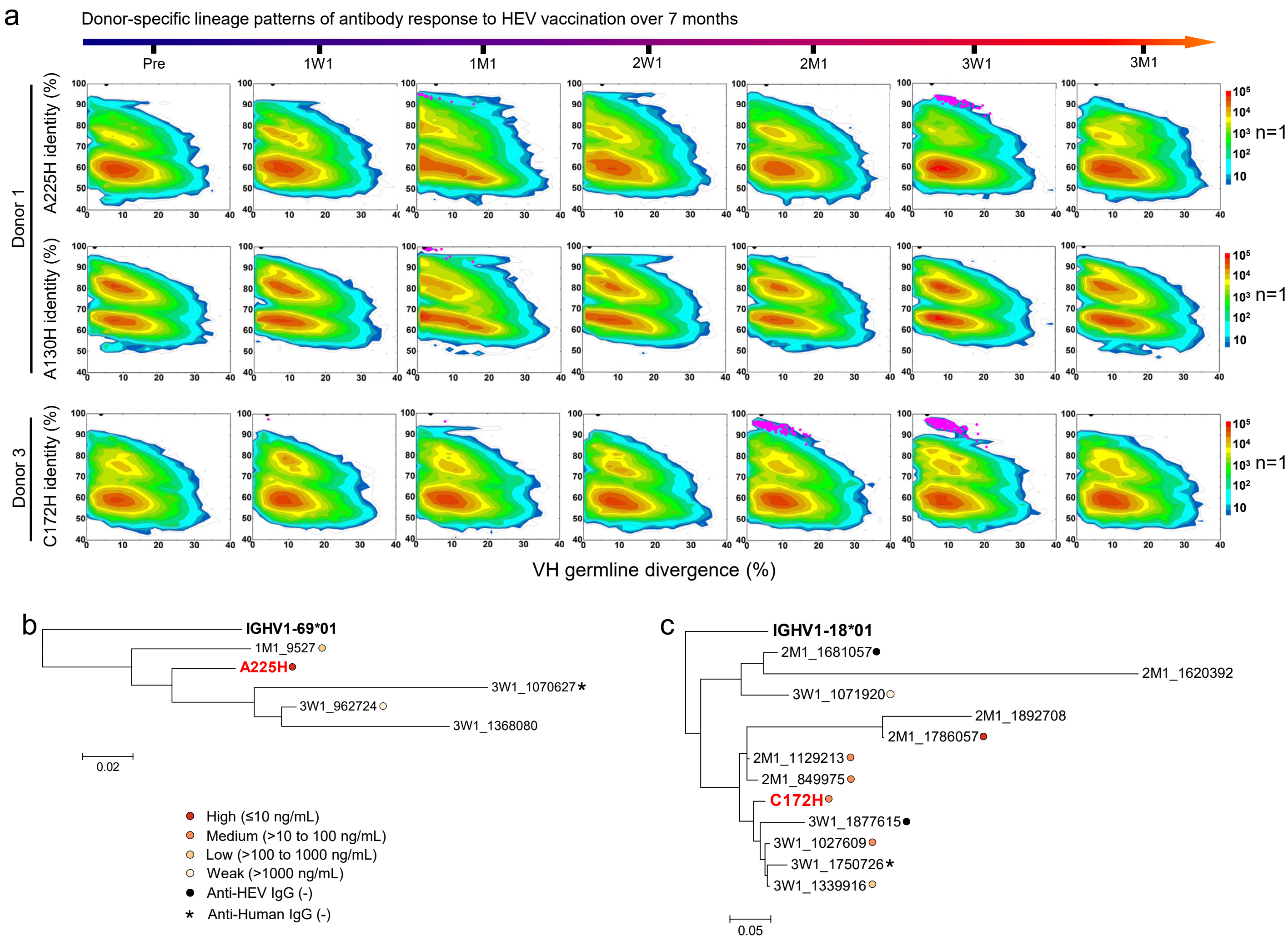

Supplementary Figure 18. Donor-specific patterns of the HEV p239(1)-specific antibody lineage development during the vaccination. (**a**) Identity-divergence plots of the A225, A130, and C172 lineages in the context of unbiased donor B cell repertoires. Heavy chains are plotted as a function of sequence identity to the reference mAbs and sequence divergence (%) from their putative germline genes. Color-coding indicates sequence density on the 2D plot. Somatic variants of A225, A130, and C172 identified by a CDRH3 identity cutoff of 90% are shown as magenta dots on the 2D plots. Pre: pre-vaccination; 1W1: one week after the first dose; 1M1: one month after the first dose; 2W1: one week after the second dose; 2M1: one month after the second dose; 3W1: one week after the third dose; 3M1: one month after the third dose. Dendrograms of selected heavy chain variants from the lineages of A225 (**b**) and C172 (**c**) rooted by their putative germline V genes. The NGS-derived somatic variants were paired with light chains of their parental mAbs for functional validation. Reconstituted mAbs are labeled with dots if they can be expressed and bind the HEV vaccine antigen, p239(1). The parental mAb heavy chains is labeled in red on the dendrogram, which was generated using the maximum-likelihood (ML) method. (EC_{50} [ng/mL]: ≤10, red circle; >10 to 100, orange circle; >100 to 1000, aurantia circle; >1000, yellow circle; p239(1)-nonspecific, black).
